# The fission yeast ortholog of Coilin, Mug174, forms Cajal body-like nuclear condensates and is essential for cellular quiescence

**DOI:** 10.1093/nar/gkae463

**Published:** 2024-06-03

**Authors:** Xiaoling Deng, Qinglian Yao, Attila Horvath, Ziling Jiang, Junjie Zhao, Tamás Fischer, Tomoyasu Sugiyama

**Affiliations:** School of Life Science and Technology, ShanghaiTech University, Shanghai 201210, China; School of Life Science and Technology, ShanghaiTech University, Shanghai 201210, China; The John Curtin School of Medical Research, The Australian National University, Canberra 2601, Australia; School of Life Science and Technology, ShanghaiTech University, Shanghai 201210, China; School of Life Science and Technology, ShanghaiTech University, Shanghai 201210, China; The John Curtin School of Medical Research, The Australian National University, Canberra 2601, Australia; School of Life Science and Technology, ShanghaiTech University, Shanghai 201210, China

## Abstract

The Cajal body, a nuclear condensate, is crucial for ribonucleoprotein assembly, including small nuclear RNPs (snRNPs). While Coilin has been identified as an integral component of Cajal bodies, its exact function remains unclear. Moreover, no Coilin ortholog has been found in unicellular organisms to date. This study unveils Mug174 (Meiosis-upregulated gene 174) as the Coilin ortholog in the fission yeast *Schizosaccharomyces pombe*. Mug174 forms phase-separated condensates *in vitro* and is often associated with the nucleolus and the cleavage body *in vivo*. The generation of Mug174 foci relies on the trimethylguanosine (TMG) synthase Tgs1. Moreover, Mug174 interacts with Tgs1 and U snRNAs. Deletion of the *mug174*^+^ gene in *S. pombe* causes diverse pleiotropic phenotypes, encompassing defects in vegetative growth, meiosis, pre-mRNA splicing, TMG capping of U snRNAs, and chromosome segregation. In addition, we identified weak homology between Mug174 and human Coilin. Notably, human Coilin expressed in fission yeast colocalizes with Mug174. Critically, Mug174 is indispensable for the maintenance of and transition from cellular quiescence. These findings highlight the Coilin ortholog in fission yeast and suggest that the Cajal body is implicated in cellular quiescence, thereby preventing human diseases.

## Graphical Abstract

**Graphical Abstract:**
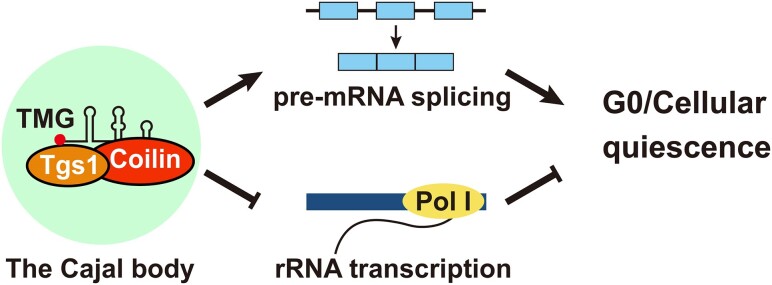

## Introduction

The nucleus is the most prominent organelle in eukaryotic cells, with numerous nuclear proteins modulating varied nuclear transactions, including chromatin assembly, transcription, and RNA processing (1). Many nuclear proteins associated with these transactions are unevenly present throughout the nucleus. Conversely, these proteins localize to specific nuclear compartments, known widely as nuclear bodies or nuclear condensates (2–4). Recent research into biological condensates has indicated that biological condensates have no membrane. Instead, phase separation directed by disordered or prion-like proteins assembles such membrane-less compartments (5–7). Moreover, the primary function of these phase-separated compartments is to increase the efficiency and specificity of specific biological processes (8,9), with dysfunction in these nuclear bodies tied to neurodegenerative disorders and cancer, demonstrating their biological and clinical importance (9–11).

The Cajal body, a class of biological condensate in the nucleus, was identified over 100 years ago and is typically associated with the nucleolus (12,13). Cajal bodies (CBs) are involved in the formation of ribonucleoproteins (RNPs), such as snRNPs and snoRNPs (4,14,15). Specifically, CBs enable snRNA transcription by clustering snRNA genes around CBs (16) and act as sites where snRNP assembly and snRNA modifications (methylation and pseudouridylation) occur (17). Additionally, CBs play an important role in nonsense-mediated mRNA decay, RNAi-based gene silencing, viral infection, and stress response (18,19). Several human diseases are associated with mutations in genes encoding CB components (4). Therefore, CBs are nuclear structures essential for various nuclear transactions in eukaryotes.

CBs contain various proteins and RNAs, with one of the most representative constituents of CBs being Coilin (20,21). Coilin is an approximately 80 kDa nuclear protein conserved in multicellular organisms, from *Trichoplax adherens* to humans. The primary sequence of Coilin is conserved at the N- and C-terminal regions: the central region of Coilin is not conserved but marked by an intrinsically disordered domain (22). In addition, Coilin is associated with many factors, including SMN (survival of motor neuron protein), the WD40 repeat-containing protein WRAP53, the nucleolar protein NOPP140, as well as RNAs (4,15). Considering the functions of CBs, Coilin disruption could trigger severe defects in cellular function and development. However, the importance of Coilin is debated (21). For instance, *Drosophila melanogaster* lacking Coilin do not exhibit any phenotypic change, while the Cajal body is severely disrupted (23), whereas 50% of Coilin knock-out mice are viable (24). Conversely, Coilin knock-down in zebrafish embryos indicates the indispensability of this protein for snRNP assembly and embryogenesis (25). These results suggest that the requirement of CBs varies depending on cell type, developmental stage, environmental condition, and organism. As Coilin has not been identified in lower eukaryotic model organisms like *C. elegans* and yeast, characterizing Coilin in *C. elegans* or yeast would be an important step forward to better understanding Coilin.

In this study, we identified fission yeast Mug174 as a nuclear protein forming biological condensates via a localization-based screen and demonstrated that Mug174 is the ortholog of Coilin in *Schizosaccharomyces pombe*. Mug174/Coilin produces phase-separated condensates *in vitro*, and Mug174 localization *in vivo* requires its N-terminal and disordered domains. Moreover, Mug174/Coilin interacts and colocalizes with the RNA trimethylguanosine synthase Tgs1/TGS1. Genetic assessments underscored Mug174/Coilin's necessity for normal cell growth, meiosis, genomic stability, U snRNA maturation, and pre-mRNA splicing. Moreover, we revealed that Mug174/Coilin is necessary for the maintenance and transition of cellular quiescence. Based on our findings, the equivalent nuclear compartment to CBs is also present in fission yeast. We propose that defective cellular quiescence as a result of Cajal body dysfunction could lead to human diseases, such as spinal muscular atrophy and dyskeratosis congenita.

## Materials and methods

### Strains and media

The genetic approaches employed in this study have been documented previously (26,27). Complete medium (YES), low adenine medium (YE), minimal medium (EMM, MSL or PMG), MSL lacking adenine (MSL-Ade), MSL lacking leucine (MSL-Leu), MSL lacking uracil (MSL-Ura), and sporulating medium (SPA) were employed (28). Moreover, deletion strains and those expressing proteins fused with GFP (29), mCherry (30), a 13 × myc epitope, a 5 × FLAG epitope, or tdTomato (30) were produced via PCR-based methods as outlined previously (31). The *S. pombe* strains utilized in this study are provided in Supplementary Table S1. All primers for strain construction are summarized in Supplementary Table S2.

### Assays using *S. pombe*

The mating efficiency of homothallic cells, sporulation efficiency of diploid cells, and mitotic minichromosome (Ch16m23) loss were examined as described previously (32–34). For growth-curve assessment, *S. pombe* strains were cultured in a complete liquid medium to mid-log phase, and the cultures were diluted to an optical density (OD_600_) of 0.02. The diluted cultures were grown at 18°C, 26°C, 32°C or 37°C, and OD_600_ readings were taken at various time points.

### Microscopic analysis

A Zeiss Axio Imager Z2 microscope (Carl Zeiss MicroImaging) was utilized for differential interference contrast (DIC) and fluorescence microscopy. The raw images were processed using ZEN lite 2012 (Carl Zeiss MicroImaging). An Olympus microscope BX53 connected to an SC180 digital camera was employed to acquire DIC images.

Regarding 1,6-hexanediol treatment, yeast cells expressing Mug174-GFP were cultured in a minimal liquid medium (untreated, UT) and then in a minimal liquid medium supplemented with 5% (w/v) 1,6-hexanediol (72210A, Adamas) for 10 minutes. Cells treated with 1,6-hexanediol (treated, T) were rinsed twice with a minimal liquid medium to eliminate residual 1,6-hexanediol. The washed cells were grown in a fresh minimal liquid medium for 20 min (recovered, R) prior to image acquisition.

To determine lagging chromosomes during the M phase, wild-type and *mug174*Δ cells expressing GFP-Atb2 (alpha-tubulin) and Hta1 (histone H2A)-mCherry were utilized. The two strains were cultured in liquid minimal media until reaching the exponential mid-log phase. Then, these strains were examined using a fluorescent microscope. The number of cells exhibiting lagging chromosomes in cells at anaphase (identified based on the length of GFP-Atb2) was determined.

### Protein purification and *in vitro* droplet formation assay

*E. coli* BL21 cells expressing the MBP (maltose-binding protein)-SNAP-Mug174-6 × His tag fusion protein were lysed via sonication in lysis buffer [50 mM Tris–HCl (pH 7.5), 1 M NaCl, 1 M urea, 10 mM imidazole, 1.5 mM 2-mercaptoethanol, 1% NP-40, 5% glycerol and protease inhibitor cocktail (P88300, ABCone)], and the fusion protein was purified using Ni NTA Beads (SA004025, Smart-Lifesciences) followed by Amylose Resin (E8021S, NEB).

For *in vitro* droplet formation assays, MBP-SNAP-Mug174-6 × His was digested using TEV protease (C500302, Sangon) for 1 hour at 30°C to remove the MBP/His-tag. Then, SNAP-Mug174 was incubated with SNAP-Cell 647-SiR (S9102S, NEB) in the dark for 30 min at 37°C. Following the reaction, the proteins were diluted in assay buffer [20 mM Tris–HCl (pH 7.4), 150 mM NaCl, and 1 mM DTT) to the indicated concentrations. The droplets produced by SNAP-Mug174 were examined using a Zeiss Axio Imager Z2 microscope (Carl Zeiss MicroImaging).

### Multi-copy suppressor screen

A heterothallic *mug174*Δ strain was transformed with a high copy number genomic DNA library (pREP-based) from the National Bio-Resource Project (NBRP), Japan. Transformants grown at 32°C were selected using MSA (28) plates lacking leucine. The candidate plasmids capable of rescuing the growth defect in *mug174*Δ were isolated from the transformants and re-transformed into the *mug174*Δ strain to confirm their ability to rescue the growth defective phenotype. The positive plasmids were sequenced to characterize the genes responsible for the rescue.

### Western blotting

Protein samples for western blotting were isolated using an alkaline extraction method (35). Anti-mCherry (PA5-34974, ThermoFisher Scientific), anti-GFP (clones 7.1 and 13.1, Roche), anti-myc (71D10, Cell Signaling Technology) and anti-His (HT501-01, Transgen) antibodies were utilized for probing epitope-tagged proteins. Western blotting was performed a minimum of two times independently. Protein loading was investigated through Direct Blue 71 staining (36).

### RT-PCR and RT-qPCR

Total RNA was isolated using the MasterPure Yeast RNA Purification Kit (MPY03100, Epicentre). DNA contamination in the RNA samples was degraded utilizing RNase-free TURBO DNase (AM2238, ThermoFisher Scientific). Complementary DNA (cDNA) was synthesized using the PrimeScript 1st strand cDNA Synthesis Kit (6110A, Takara Bio). Negative controls were performed in reaction mixtures without reverse transcriptase. Target DNA fragments were amplified using a T100 Thermal Cycler (Bio-Rad). The amplified fragments were combined with Agilent DNA 1000 Reagents (5067–1505, Agilent) and examined using a 2100 Electrophoresis Bioanalyzer Instrument (G2939AA, Agilent). For RT-qPCR, cDNA sample preparation mirrored the procedure for RT-PCR. qPCR was performed using ChamQ Universal SYBR qPCR Master Mix (Q711-03, Vazyme) with a QuantStudio 7 Flex (ThermoFisher Scientific). The primers utilized in RT-PCR and RT-qPCR are documented in Supplementary Table S2.

### RNA sequencing

Total RNA samples employed for RNA-Seq were extracted using the same method outlined in the RT-PCR section. Strand-specific RNA sequencing libraries were produced using poly(dT)-enriched RNAs isolated from wild-type (WT) and *mug174*Δ alongside the NEBNext Ultra II RNA Library Prep Kit for Illumina (E7530L, NEB) following the manufacturer's directions. The raw data were processed as outlined in the prior study (37). Specifically, paired-end RNA-seq reads were aligned to the *S. pombe* reference genome (ASM294v2, https://www.pombase.org/) using HISAT2 (38), permitting a maximum intron length of 2000 bp. Alignment files were sorted and indexed utilizing SAMtools. Intronic reads were filtered using the Rsamtools and GenomicAlignments R packages. Bigwig files were produced with bamCoverage from deepTools with 1-bp resolution.

Transcripts that increased >2^0.6^ (approximately equal to 1.5-fold) or decreased <2^−0.6^ (approximately equal to 0.65-fold) were transcripts with expression level alterations. The total number of increased and decreased mRNAs were 251 and 383, respectively. Additionally, the total number of increased and decreased ncRNAs were 344 and 94, respectively. The increased and decreased genes are highlighted in Supplementary Tables S4 and S5.

Gene ontology (GO) analysis was performed by AnGeLi (39). The sequencing read coverage was visualized using the Integrative Genomics Viewer (IGV). The statistical significance (*p*-value) of the overlap between the two groups was identified through the hypergeometric probability distribution.

### Chromatin immunoprecipitation

Chromatin immunoprecipitation (ChIP) was conducted as outlined previously (40) with slight modifications. Specifically, fission yeast cells were grown to 1–2 × 10^7^ cells/mL (OD_600_ = 0.5–1.0) in a complete liquid medium followed by fixation with 3% (v/v) formaldehyde (M48418030, Macklin) at room temperature for 30 min. Cell lysates containing sheared chromatin were incubated with an anti-H3K9me2 antibody (Ab1220, Abcam), an anti-myc antibody (71D10, Cell Signaling Technology), or an anti-FLAG antibody (F1804, Sigma Aldrich) at 4°C overnight. Precipitated chromatin was treated with RNase/proteinase K, and DNA was purified using the NucleoSpin Gel and PCR Clean-UP kit (740609.250, MACHEREY-NAGEL) to isolate template DNA for qPCR. The primers employed in ChIP-qPCR are outlined in Supplementary Table S2.

### RNA-FISH and immunostaining

RNA-FISH was conducted as described previously (41). Specifically, yeast cells grown to the logarithmic growth phase were fixed using 4% paraformaldehyde, treated with 300 U/mL lyticase (L2524, Sigma-Aldrich), and incubated with 5′-Cy3 labeled probes overnight to detect U1/U2/U5 snRNAs. The oligonucleotide probes employed in RNA-FISH are outlined in Supplementary Table S2. To perform immunostaining, the spheroplasts were subjected to an overnight incubation with anti-2,2,7-trimethylguanosine antibody (RN019M, MBL) at 4°C, followed by a one-hour incubation with a goat anti-mouse IgG (H + L) cross-adsorbed secondary antibody, conjugated to Alexa Fluor 594 (A-11005, ThermoFisher Scientific) at room temperature. The DNA was counterstained using DAPI before imaging.

### Northern blotting

Small RNAs were extracted from fission yeast cells using RNAiso for Small RNA (9753Q, TaKaRa Bio). The purified small RNAs were separated using urea-containing denatured polyacrylamide gels (R0235S, Beyotime) with TBE buffer. The small RNAs in the gels were electro-transferred to a nylon membrane (77015, ThermoFisher Scientific) and underwent UV cross-linking. The membrane was pre-hybridized at 40°C in pre-hybridization buffer [200 mM Na_2_HPO_4_ (pH 7.0), 7% SDS, 5 μg/mL salmon sperm DNA] at 40°C for 3 h and then hybridized with 50 nM biotin-labeled probes in hybridization buffer at 40°C for 16 h. The membrane was rinsed three times with 1 × SSC (15 mM sodium citrate, 150 mM NaCl) with 0.1% SDS at room temperature. A Chemiluminescent Nucleic Acid Detection Module Kit (89880, Thermo Scientific) was employed to detect biotin-labeled probes. The oligonucleotide probes used in northern blotting are listed in Supplementary Table S2.

### RNA immunoprecipitation

RNA immunoprecipitation (RNA-IP) was performed as previously delineated (32). Specifically, *S. pombe* cells grown to the logarithmic growth phase were resuspended in RNA-IP buffer, composed of 50 mM HEPES (pH 7.5), 140 mM NaCl, 10% glycerol, 1 mM EDTA, 0.1% Triton X‐100, 0.1% NP‐40, 1 mM PMSF, 2 mM vanadyl ribonucleoside complex and 400 U/ml RNasin Plus RNase inhibitor. The cells were disturbed using bead-beating. The cleared cell extracts were mixed with anti-GFP antibodies (ab290, Abcam) and protein A/G magnetic beads (B23201, Bimake) at 4°C for 3 h. The magnetic beads were rinsed three times with RNA-IP buffer, and the precipitated RNA was extracted using Trizol (Sangon). After digesting the residual DNA, the precipitated RNA was subjected to RT-qPCR.

RNA-IP utilizing an anti-trimethyl guanosine (TMG) cap antibody was conducted as outlined previously (42). Essentially, DNase-treated total RNA samples were incubated with anti-TMG antibodies (RN019M, MBL) and protein A/G magnetic beads (B23201, Bimake) at 4°C overnight. After bead rinsing, the precipitated TMG-capped RNA was extracted and used for RT-qPCR. The primers employed for RT-qPCR are outlined in Supplementary Table S2.

### Yeast-two hybrid assay

The coding sequences corresponding to investigated proteins were amplified via PCR using *S. pombe* genomic DNA and PrimeSTAR Max DNA Polymerase (R045Q, TaKaRa Bio) before being cloned into the pGAD-T7 or pGBK-T7 vector for utilization in yeast two-hybrid (Y2H) assays with the NovoRec plus One step PCR Cloning Kit (NR005, Novoprotein). The resultant plasmids with protein-coding sequences were employed to transform the *Saccharomyces cerevisiae* strain AH109 (YC1010, Shanghai Weidi Biotechnology). The transformants were selected on synthetic minimal medium plates (SD) without leucine and tryptophan (SD-Leu-Trp). A 10-fold serial dilution of the transformants was plated onto SD-Leu-Trp, SD-Leu-Trp-His or SD-Leu-trp-His-Ade media, and incubated at 30°C for 3 days. The combination of pGAD-T7-large T antigen and GBK-T7-p53 was employed as a positive control, while pGAD-T7-T antigen and pGBK-T7-Lamin served as a negative control. All primers used in plasmid construction are outlined in Supplementary Table S2. The plasmids utilized for Y2H are documented in Supplementary Table S3.

### Immunoprecipitation

*S. pombe* cells grown to the mid-log phase were lysed in 200 μl of ice-cold lysis buffer [10 mM Tris–HCl (pH 7.5), 150 mM NaCl, 0.5 mM EDTA, 0.5% NP40, 2.5 mM MgCl_2_, and 1 mM PMSF] supplemented with protease inhibitors (P88300, ABCone) and diluted by adding 300 μl of dilution buffer [10 mM Tris–HCl (pH 7.5), 150 mM NaCl and 0.5 mM EDTA]. After centrifugation, cleared lysates were mixed with anti-GFP beads (SB-NM001, ShareBio) at 4°C for 2 h, followed by three washes with 1 × PBS supplemented with 0.1% Tween 20. Precipitated proteins were separated by SDS-PAGE followed by western blotting.

### Proteome analyses

Protein samples were prepared as outlined previously (43). Specifically, cells grown to the exponential phase were lysed in lysis buffer, including 100 mM ammonium bicarbonate, 8 M urea, and protease inhibitors (P88300, ABCone). Cell lysates were sonicated using a Bioruptor Plus (Diagenode). After measuring the protein concentration in each extract, the samples were subjected to mass spectrometry. The protein samples were digested using trypsin (Promega) and subjected to desalting using a C18 StageTip. Liquid chromatography with tandem mass spectrometry (LC–MS/MS) was conducted with a Q-Exactive HF-X mass spectrometer linked to Easy 1200 nLC (Thermo Fisher Scientific). The MS data were investigated using the Proteome Discoverer software version 2.4. MS analysis was replicated twice independently.

### G0 experiments

Heterothallic (*h*^−S^) prototroph fission yeast cells were grown in liquid EMM, rinsed three times with sterilized water, and grown in liquid EMM-nitrogen (EMM-N) to induce G0 entry. To assess G0 entry, cells were rinsed with sterilized water and fixed with 70% ethanol at 4°C overnight. The fixed cells were rinsed in 50 mM sodium citrate, treated with RNase A, and stained with propidium iodine. The stained samples were investigated using flow cytometry.

At various time points (0, 1, 7, 14, 21 or 28 days following nitrogen deprivation), an aliquot of each culture was subjected to trypan blue exclusion testing to characterize cell viability within G0. At the corresponding time points, 90 or 182 cells in G0 were placed onto complete medium plates with a dissecting microscope (MSM400, Singer Instruments) to characterize mitotic competence, referring to the ability to reverse the mitotic cell cycle from G0.

## Results

### Mug174 forms nuclear foci

**Figure 1.**
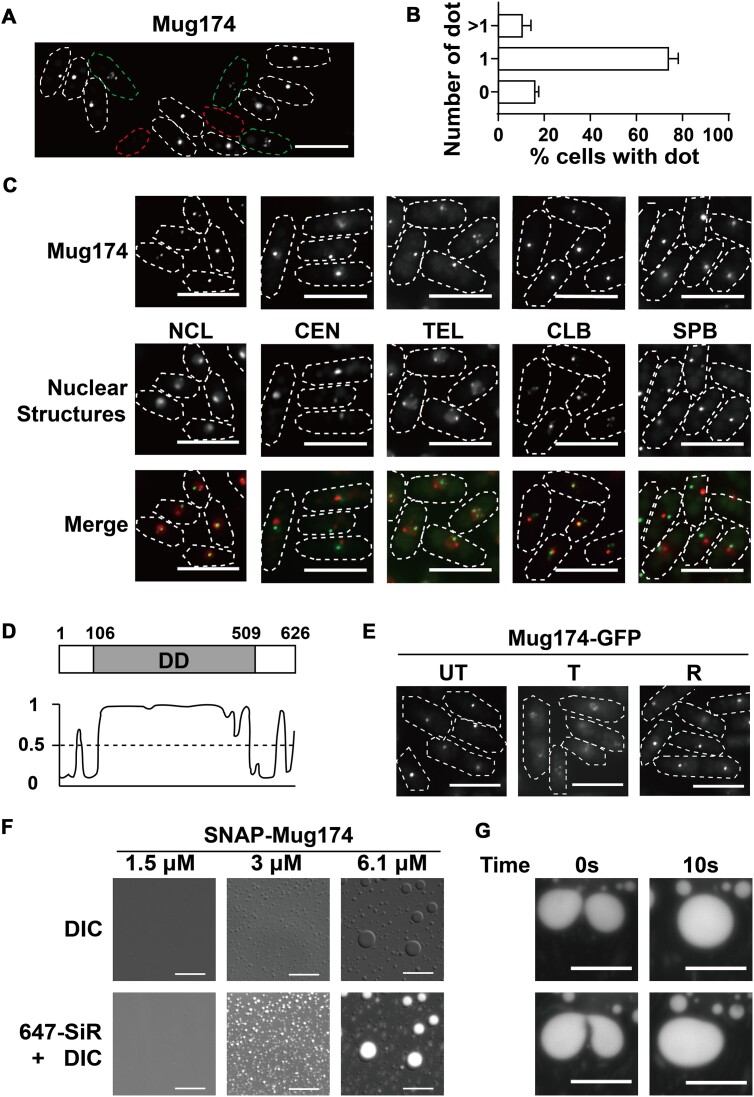
Mug174 forms nuclear foci via phase separation. (**A**) Localization of Mug174-GFP in vegetative cells. The dotted lines indicate the cell shapes. Red dotted line: no Mug174 dot, white dotted line: one Mug174 dot, green dotted line: more than one Mug174 dot. Scale bars, 10 μm. (**B**) The percentage of cells containing 0, 1 or >1 Mug174 focus. Over 100 cells were examined each time, and the mean ± S.D. was presented (*n* = 3). (**C**) Localization of Mug174 and five known nuclear structures in *S. pombe*. Three strains expressing Mug174-GFP and one Gar2-mCherry (the nucleolus; NCL), Cnp3-tdTomato (centromeres; CEN), and Taz1-tdTomato (telomeres; TEL), or two stains expressing Mug174-tdTomato with either Red1-GFP (the cleavage body; CLB) or Sad1-GFP (spindle pole body; SPB). The white dotted lines indicate the cell shapes. Scale bars, 10 μm. (**D**) A prediction of the intrinsically disordered domain (DD) in the Mug174 protein using DISOPRED3. A cutoff level of 0.5. (**E**) Mug174-GFP localization before (untreated; UT), during (treated; T), and after (recovery; R) 1,6-hexanediol treatment. White dotted lines indicate cell shapes. Scale bars, 10 μm. (**F**) Microscopy images (DIC and DIC merged with fluorescent signals) of the spherical structures produced by SNAP-Mug174 at 3 μM and 6.1 μM. SNAP-Mug174 was labeled using SNAP Cell 647-SiR. Scale bars, 20 μm. (**G**) The spherical structures produced by SNAP-Mug174. Two structures began fusion at an arbitrary time point (0 seconds) and became a larger structure within 10 s. Scale bars, 20 μm.

We previously performed a localization-based screen of proteins exhibiting specific localization patterns using *S. pombe* (32,33,44,45). In the same identification procedure, we identified that Mug174, encoded by a meiotically upregulated gene (46), formed discrete foci within the nucleus during vegetative growth (Supplementary Figure S1A). A majority of cells exhibited one Mug174 dot, while others had more than one dot or no dots (Figure 1A and B). Our findings are consistent with another study reporting Mug174 localization (47). A previous large-scale deletion experiment indicated that *S. pombe* cells lacking Mug174 generate abnormal spores (48), however, the functions of Mug174 remained unexamined. Therefore, we investigated Mug174, aiming to find a previously unidentified nuclear condensate.

A standard BLAST search did not find homologs in higher eukaryotes, while Mug174 is conserved across other fission yeast species (Supplementary Figure S1B). We investigated whether Mug174 is a component of previously identified nuclear structures. We combined Mug174-GFP with several marker proteins and investigated their localization. As illustrated in Figure 1C and Supplementary Figure S1C, Mug174 did not colocalize with centromeres (CEN), telomeres (TEL), or the spindle pole body (SPB, equivalent to the centrosome in higher eukaryotes). Notably, we observed that Mug174 was present in close proximity to the nucleolus and colocalized with the cleavage body (CLB, containing pre-mRNA 3′ processing factors) (Figure 1C and Supplementary Figure S1D). These results suggest that: (1) Mug174 is situated in a previously unrecognized nuclear structure in *S. pombe* and (2) the Mug174-containing nuclear structure may have a functional connection to the nucleolus and the cleavage body.

### Mug174 can direct liquid–liquid phase separation

Recent progress in biological condensate research has suggested that biological condensate assembly relies on phase separation by proteins with intrinsically disordered or prion-like domains (5,6). We examined whether Mug174 possessed any intrinsically disordered or prion-like domains. The primary sequence of Mug174 was subjected to analyses using DISOPRED3 (49) and PLAAC (50). DISOPRED3 predicted a disordered structure within the central portion of Mug174 (Figure 1D), while PLAAC did not identify any prion-like domain in Mug174 (data not shown). We then tested whether Mug174 foci are sensitive to 1,6-hexanediol (1,6-HD), a widely utilized aliphatic chemical that interferes with phase separation (51,52). We determined that Mug174 foci disappeared upon 1,6-HD treatment and reappeared upon 1,6-HD removal (Figure 1E). This disassembly and reassembly of Mug174 foci was not associated with any alterations in Mug174 protein levels (Supplementary Figure S1E), suggesting that it forms nuclear foci through phase separation, exhibiting dynamic foci assembly. To confirm that Mug174 can assemble phase-separated condensates, we expressed MBP-SNAP-Mug174-6 × His (Supplementary Figure S1F) and purified the fusion protein (Supplementary Figure S1G), followed by an *in vitro* droplet formation assay using recombinant SNAP-Mug174 (Supplementary Figure S1G). Solutions of SNAP-Mug174 (at 3 and 6.1 μM, but not 1.5 μM) included spherical droplets emitting fluorescent light at room temperature under a physiological salt condition (150 mM NaCl) (Figure 1F). The spherical droplets migrated freely in solutions and occasionally combined (Figure 1G and Supplementary Movie S1). These results support the idea that Mug174 can undergo liquid-liquid phase separation to produce nuclear condensates.

### The Mug174 N-terminal and disordered domains are required for proper localization

To further examine the formation of Mug174 condensates *in vivo*, we produced strains expressing GFP-fused Mug174 lacking each domain (Mug174ΔN, ΔDD or ΔC) from the endogenous *mug174*^+^ locus. However, we did not identify GFP signals via western blotting or under a microscope except for Mug174ΔC-GFP (data not shown), suggesting that both the N-terminal and disordered domains are required for the integrity of the protein *in vivo*. We constructed six Mug174-GFP expression plasmids (full-length, ΔN, ΔDD, ΔC, N and DD) to express Mug174 proteins heterologously (Supplementary Figure S1H). Western blotting indicated that all GFP-fusion proteins were expressed at noticeable levels (Supplementary Figure S1I and J). We examined the localization of each GFP-fusion protein, finding that Mug174ΔC and full-length Mug174, but not ΔN, ΔDD, N or DD, produced nuclear condensates (Supplementary Figure S1K). Moreover, ΔN and DD were enriched in the nucleus relative to the cytoplasm (Supplementary Figure S1K), indicating that the disordered domain harbors a nuclear localization signal. To support this idea, the Eukaryotic Linear Motif (ELM) resource (53) found a potential nuclear localization signal (464 RRKKARI 470) in DD. Based on these observations, we determined that both the N-terminal and disordered domains are necessary for appropriate localization, as well as protein expression.

### The absence of Mug174 leads to growth hindrances

**Figure 2.**
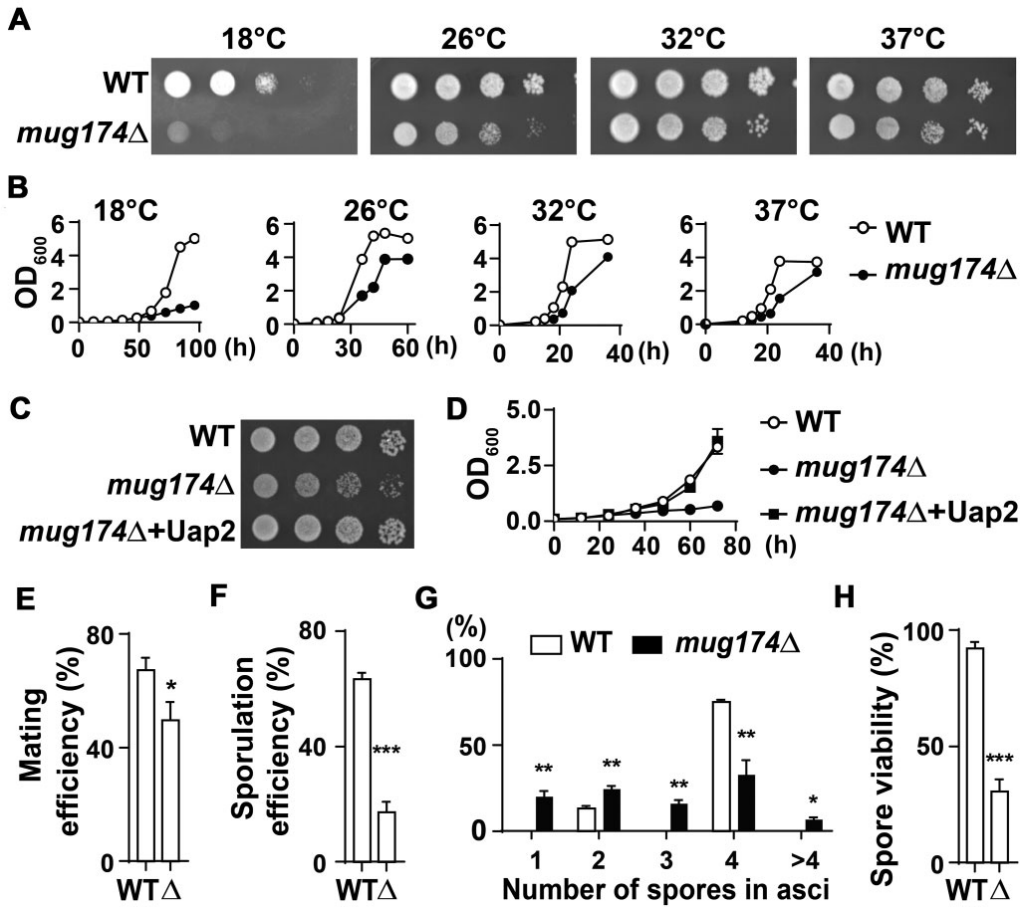
Loss of Mug174 causes mitotic and meiotic defects. (**A**) Growth of wild-type (WT) and *mug174*Δ cells at varying temperatures. Ten-fold serial dilutions were placed onto complete medium plates and incubated at the indicated temperatures. (**B**) Growth curves of WT and *mug174*Δ at various temperatures. Both strains were grown in complete liquid media, and OD_600_ was examined at the indicated time points. (C and D) *uap2^+^* suppresses the growth defect of *mug174Δ*. Dilution analysis (**C**) and the growth curve (**D**) of WT and *mug174*Δ harboring an empty plasmid (WT and *mug174*Δ), or *mug174*Δ carrying the *uap2*^+^ expression plasmid (*mug174*Δ+Uap2). The denoted cells were grown at 32°C. (**E**) *mug174*Δ displays lower mating efficiency relative to WT. Meiosis was induced in homothallic WT and *mug174*Δ strains, with mating efficiency calculated. A total of 500 cells were investigated in each replicate, and the mean ± S.D. was presented (*n* = 3). * *P* < 0.05. (**F**) *mug174*Δ displays lower sporulation efficiency relative to WT cells. Diploid WT and *mug174*Δ cells were sporulated on nitrogen-limited solid media, and sporulation was investigated using a microscope. A total of 500 asci were assessed in each replicate, and the mean ± S.D. was presented (*n* = 3). *** *P* < 0.001. (**G**) The number of spores in an ascus derived from homothallic WT and *mug174*Δ cells. Over 200 asci were investigated for each strain, and the mean ± S.D. was presented (*n* = 3). * *P* < 0.05 and ** *P* < 0.01. (**H**) The viability of spores derived from homothallic WT and *mug174*Δ cells. A total of 162 spores from each strain were assessed, and the mean ± S.D. was presented (*n* = 3). *** *P* < 0.001.

To examine Mug174 functions, we deleted *mug174*^+^ and began characterizing *mug174*Δ by examining vegetative growth. We noted that *mug174*Δ cells produced smaller colonies on tetrad-dissected plates compared to WT cells (data not shown). Dilution analysis of WT and *mug174*Δ suggested that *mug174*Δ grew slowly relative to WT, and this growth hindrance was more pronounced at 18°C (Figure 2A). We also performed growth curve analyses and found that *mug174*Δ grew slowly at all investigated temperatures (Figure 2B), indicating that Mug174 is necessary for normal cell growth, but it is not essential for cell viability.

To disentangle the growth defect resulting from *mug174*Δ, we conducted a screen for multi-copy suppressors using an *S. pombe* genomic DNA library. We examined over 120 000 transformants and obtained 12 plasmids that strongly or weakly suppressed the growth hindrance phenotype of *mug174*Δ (Supplementary Figure S2A). From these plasmids, we focused on the plasmid including *uap2*^+^ alongside three adjacent genes (Supplementary Figure S2A). Uap2/HTATSF1 is a U2 snRNP-associated protein interacting with the U2AF small subunit Prp2/U2AF-59 and the U2 snRNP-associated protein Prp10/SF3B1 (54–57). We confirmed that *uap*2^+^ overexpression alone is sufficient to suppress the growth hindrance caused by *mug17*4Δ (Figure 2C and D). These findings indicate that Mug174 may be functionally associated with pre-mRNA splicing.

### Meiotic defects in *mug174***Δ**

Based on previous studies (46,48), Mug174 is likely essential for meiosis. To obtain additional insights into Mug174 and its utility in meiosis, we examined the localization of Mug174 during meiosis. We identified the Mug174 foci in nitrogen-starved rounded cells, cells in meiotic metaphase, and spores (Supplementary Figure S2B), suggesting that Mug174 foci persist throughout meiosis. We found both mating and sporulation efficiencies were significantly reduced in *mug174*Δ relative to WT (Figure 2E and F). Mirroring these results, homothallic *mug174*Δ cells exhibited a light iodine staining pattern relative to homothallic wild-type cells (Supplementary Figure S2C, top). These findings indicate that mating, alongside sporulation, is adversely influenced by the absence of Mug174. We observed abnormal asci (Supplementary Figure S2C, bottom) as reported previously (48) and counted the number of spores in asci. The majority of asci from WT contained four spores, while those from *mug174*Δ had less than or more than four spores (Figure 2G), suggesting defective meiotic chromosome segregation in *mug174*Δ. Additionally, *mug174*Δ produced significantly more inviable spores than WT (Figure 2H and Supplementary Figure S2D). From these findings, we can conclude that Mug174 is essential for producing viable progeny.

### Deletion of *mug174*^+^ alters the transcriptome landscape

We conducted transcriptome analyses of vegetative WT and *mug174*Δ cells to characterize genes with altered expression levels without Mug174. Two independent RNA sequencing experiments found 595 upregulated transcripts (cutoff: 2^0.6^, equal to approximately 1.5) in vegetative *mug174*Δ relative to WT cells (Supplementary Figure S3A and Supplementary Table S4). Of these, approximately 42% of the increased transcripts (251) were mRNAs, and the remainder (344) were non-coding RNAs (Supplementary Figure S3B). Conversely, we found 477 downregulated transcripts (cutoff: 2^−0.6^, equal to approximately 0.66) in vegetative *mug174*Δ compared to WT cells (Supplementary Figure S3A and Supplementary Table S5). In contrast to the upregulated transcripts, approximately 80% of the decreased transcripts (383) were mRNAs, and the remainder (94) were non-coding RNAs (Supplementary Figure S3C).

To classify the increased and decreased genes based on their functions, we conducted gene ontology (GO) analyses. The GO annotations of the significantly increased transcripts (*P* < 10^−4^ to 10^−2^) were associated with meiosis (Supplementary Table S6). The disrupted expression of meiosis-related genes in *mug174*Δ could account for the meiotic defects identified in *mug174*Δ (Figure 2E–H and Supplementary Figure S2C and D). Conversely, the GO annotation of the significantly decreased genes (*P* < ∼10^−60^) was predominantly cytoplasmic translation (Supplementary Table S6). Additionally, the GO annotations with relatively low significance (*P* < ∼10^−8^) were related to cellular metabolism (Supplementary Table S6). Based on these GO annotations, Mug174 plays a role in the correct expression of specific classes of genes directly or indirectly.

To investigate whether the transcriptome alterations produce proteome changes in *mug174*Δ, we conducted whole-cell proteome analyses of WT and *mug174*Δ cells. The mass-spectrometry data found 149 increased (cutoff: 2^0.6^) and 349 decreased (cutoff: 2^−0.6^) proteins in vegetative *mug174*Δ (Supplementary Figure S3D and Supplementary Tables S7 and S8). Notably, 70% of the increased proteins are derived from intron-less genes, while 66% of the decreased proteins are derived from intron-containing genes (Supplementary Figure S3E). This finding indicates that gene expression of intron-containing genes relies on Mug174 more than intron-less genes. We identified that decreased or increased proteins were involved in specific metabolic processes (Supplementary Table S9). We also found that the expression level of Uap2 was lowered in *mug174*Δ (Supplementary Figure S3D), suggesting that this reduction in Uap2 expression caused growth limitation in *mug174*Δ. Ultimately, we compared the elevated or reduced mRNAs and proteins in *mug174*Δ, identifying that the overlap between the two groups was statistically significant (Supplementary Figure S3F and Supplementary Table S10). Therefore, changes in mRNA levels, at least partly, account for alterations in protein levels in *mug174*Δ.

### mRNA splicing does not occur correctly in *mug174*Δ

**Figure 3.**
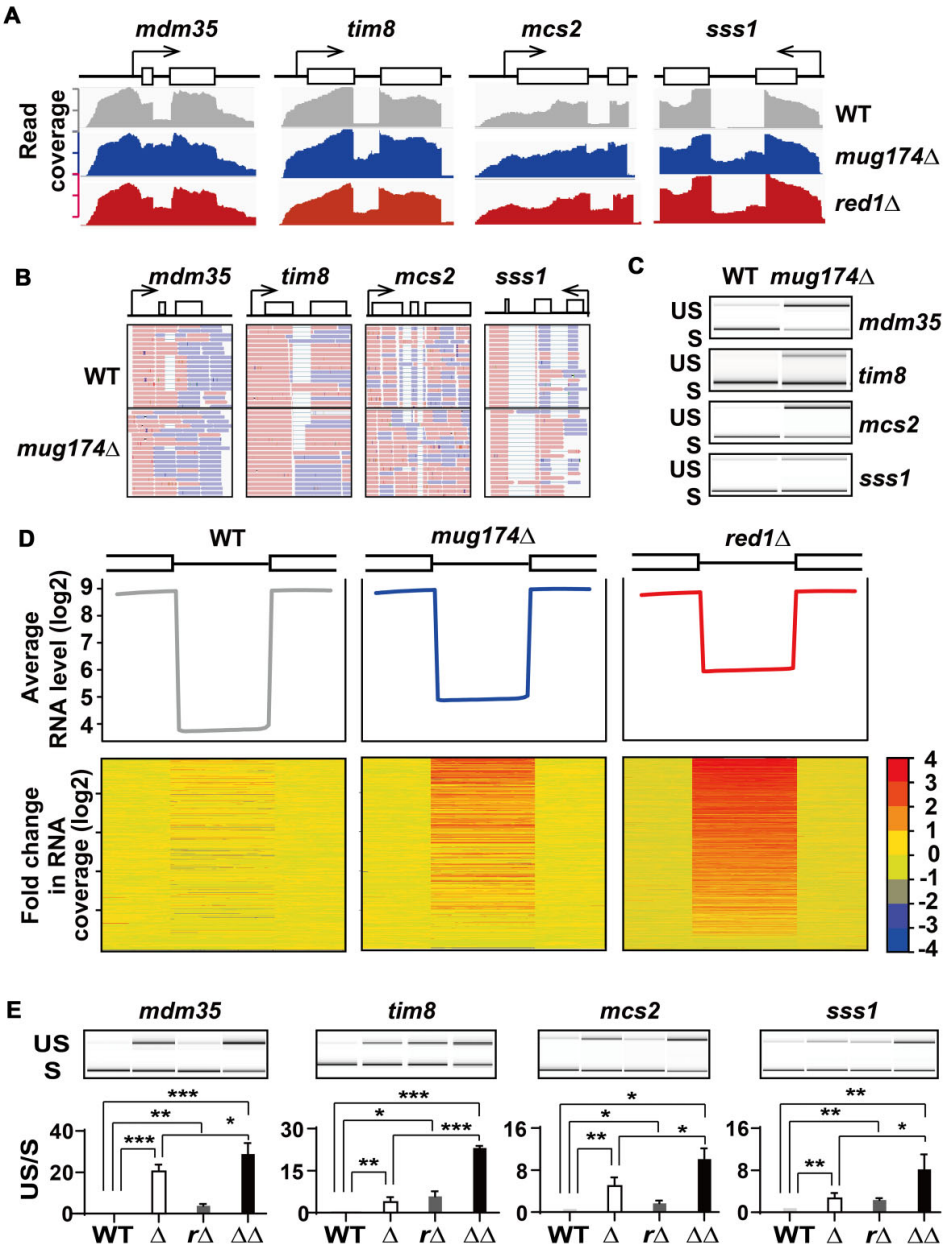
Accumulation of unspliced mRNAs in *mug174*Δ. (**A**) Strand-specific RNA-seq read coverage of indicated genes derived from wild-type (WT), *mug174*Δ and *red1*Δ cells. (**B**) Strand-specific RNA-seq reads of four representative intron-containing genes in WT and *mug174*Δ cells. Blue and red lines denote the 1st and 2nd pair reads, respectively. (**C**) RT-PCR findings of the exon-exon junctions in WT and *mug174*Δ strains. US: unspliced and S: spliced transcripts. (**D**) (top) A composite representation of RNA-seq read coverage encompassing all introns across genes (4915 introns). Each intron was separated into 30 segments (bins), and the average RNA sequence read coverage (only sense transcripts) was investigated for each bin. The surrounding 20-bp exonic regions were also included. In the resultant matrix, each row denotes an intron, and each column represents the position of the intronic region (flanking exonic regions and bins 1–40). The average of the log_2_ values (geometric average) was computed for each column and plotted along a log_2_ scale. (bottom) The heatmaps of impacted introns throughout WT, *mug174*Δ, and *red1*Δ. RNA-seq data were processed as outlined above, while alterations in intron reads (up indicated in red; down indicated in blue) were determined by comparing reads in *mug174*Δ and in *red1*Δ to those in WT. The introns of all the heatmaps were aligned from the most elevated (top) to the most reduced (bottom), according to the *red1*Δ/WT result. (**E**) RT-PCR results of the exon-exon junctions in *mug174*Δ (Δ), *red1*Δ (*r*Δ), *mug174*Δ*red1*Δ (ΔΔ) and their parental WT strains. (top panels) The lower bands indicate spliced (**S**) transcripts, while the upper bands are unspliced (US) transcripts of the indicated genes. (bottom panels) The spliced (S) and unspliced (US) mRNAs of the four representative mRNAs were quantified (mean ± S.D., *n* = 3). * *P* < 0.05, ** *P* < 0.01 and *** *P* < 0.001.

Given that our multi-copy suppressor screening and gene expression profiling indicated the possible role of Mug174 in mRNA splicing (Figure 2C and D and Supplementary Figure S2A and S3E), we investigated this possibility in our RNA-sequencing data. Intron reads were accumulated in *mug174*Δ (Figure 3A), due to increases in both intron and exon-intron junction reads (Figure 3B), with RT-PCR confirming the increases in unspliced transcripts from four representative genes (Figure 3C), demonstrating a pre-mRNA splicing defect. Our quantification of spliced and unspliced mRNAs indicated that the level of the unspliced mRNAs increased in *mug174*Δ, while the total amount of spliced and unspliced mRNA did not change substantially (Supplementary Figure S3G). Furthermore, proteome analyses demonstrated that the steady-state levels of the Mdm35, Tim8, and Msc2, but not Sss1, proteins decreased in *mug174*Δ (Supplementary Figure S3H). These findings suggest that Mug174 facilitates pre-mRNA splicing but not transcription, and the splicing defect lowers the protein expression levels, at least in some situations.

MTREC, a complex targeting various RNAs for RNA degradation by the nuclear exosome, plays a critical role in the removal of unspliced or misspliced mRNAs (37,58). During RNA-sequencing analyses, we identified that many of the introns elevated in *mug174*Δ were also increased in cells lacking the MTREC component Red1 (Figure 3A and D, bottom). These observations raised the possibility that Mug174 works alongside MTREC to enhance the degradation of unspliced mRNAs. To investigate this possibility, we examined whether Mug174 and Red1 target the same introns. The heatmap of accumulated (red) and reduced (blue) introns indicated that Mug174-target introns mirrored Red1-target introns, while the effect of *mug174*Δ on introns was not as strong as *red1*Δ (Figure 3D). Additionally, RT-PCR suggested that more unspliced mRNAs were present in *mug174*Δ*red1*Δ than in the other three strains (Figure 3E). Collectively, these findings suggest that Mug174 and Red1 suppress unspliced mRNAs via pre-mRNA splicing and degradation of unspliced mRNAs, respectively.

### Mug174 is the fission yeast ortholog of Coilin, an essential component of Cajal bodies

Our characterization of Mug174 identified that: (1) Mug174 has an intrinsically disordered domain and can form phase-separated condensates, (2) Mug174 foci often associate with the nucleolus and cleavage bodies (CLBs), (3) the N-terminal domain of Mug174 is essential for its localization, (4) Mug174 is necessary for sexual reproduction, and (5) Mug174 is involved in mRNA splicing. These findings reminded us of Coilin, an integral component of Cajal bodies (CBs) in higher eukaryotes (20,21). However, no Coilin ortholog had been reported in unicellular eukaryotes previously. We hypothesized that Mug174 operates as the fission yeast ortholog of Coilin, and we conducted additional experiments to confirm that Mug174 is the fission yeast Coilin.

**Figure 4.**
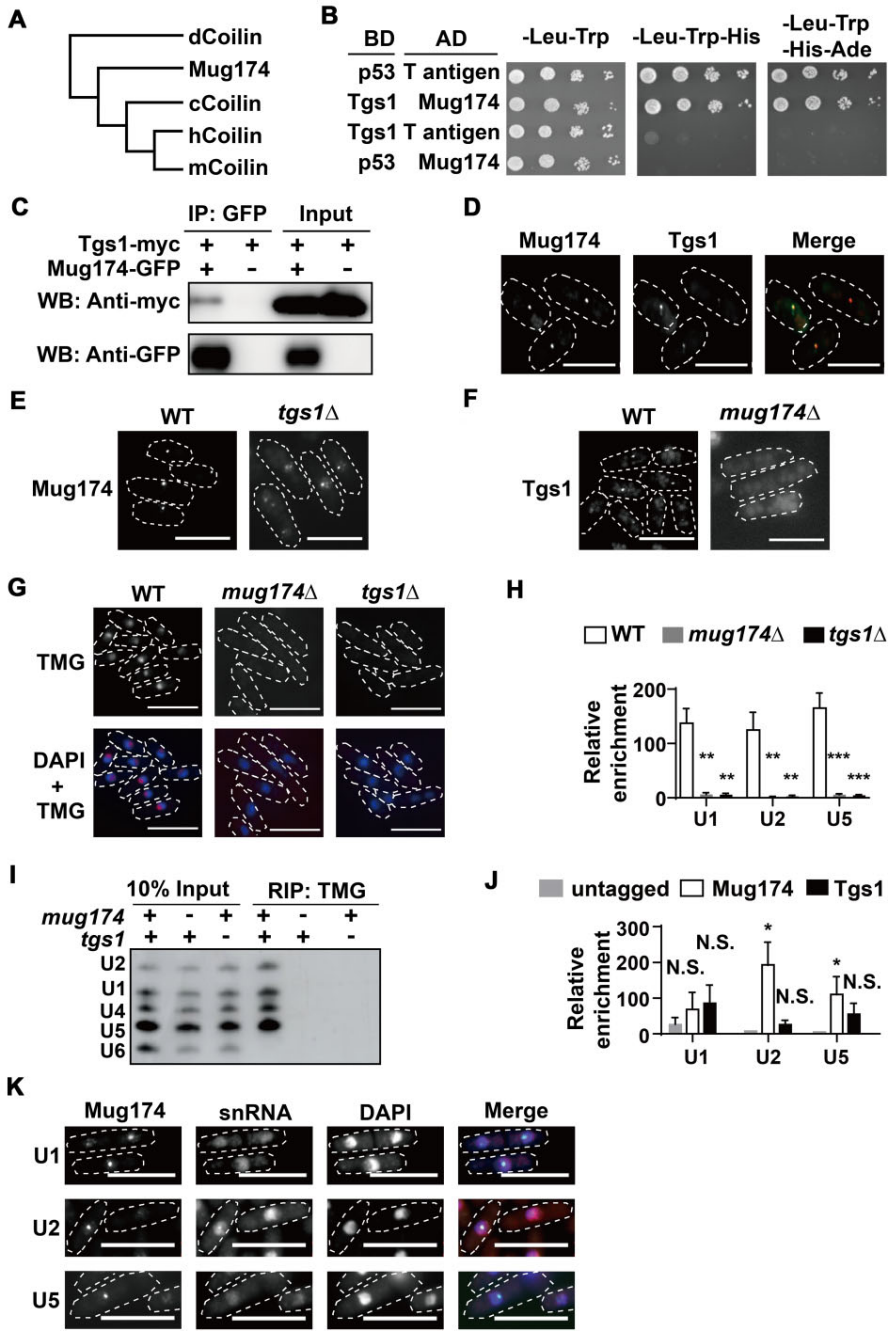
Mug174 is the Coilin ortholog in *S. pombe*. (**A**) A protein similarity tree indicating the relationship across Coilin proteins in various eukaryotic species. c: Chick, d: *Drosophila*, h: human, and m: mouse. (**B**) The interactions between Mug174 and Tgs1 were investigated using a standard yeast two-hybrid system. Mug174 was linked to the GAL4 activation domain (AD), while Tgs1 was fused to the GAL4 binding domain (BD). The combination of p53/T antigen operated as a positive control. (**C**) Tgs1 co-immunoprecipitated with Mug174. An anti-GFP antibody was subjected to immunoprecipitation to strains expressing Tgs1-myc or Mug174-GFP/Tgs1-myc. The precipitated protein samples were examined via western blotting using an anti-GFP or anti-myc antibody. (**D**) Localization of Mug174-tdTomato and Tgs1-GFP during vegetative growth. The white dotted lines denote the cell shapes. Scale bars, 10 μm. (**E**) Mug174 localization depends upon Tgs1. Wild-type (WT) and *tgs1*Δ cells expressing Mug174-GFP were assessed via fluorescence microscopy. The white dotted lines denote the cell shapes. Scale bar, 10 μm. (**F**) Tgs1 localization relies on Mug174. WT and *mug174*Δ cells expressing Tgs1-GFP were assessed by fluorescence microscopy. The white dotted lines indicate the cell shapes. Scale bars, 10 μm. (**G**) Immunofluorescence utilizing an anti-trimethylguanosine (TMG) antibody to visualize TMG in WT, *mug174*Δ and *tgs1*Δ. The white dotted lines indicate the cell shapes. Scale bars, 10 μm. (**H**) TMG caps on U1, U2 and U5 snRNAs were investigated by RNA immunoprecipitation using an anti-TMG antibody in WT, *mug174*Δ and *tgs1*Δ cells. Relative enrichment of U1, U2, and U5 snRNAs with *act1* RNA was computed and presented as mean ± S.D. (*n* = 3). ** *P* < 0.01 and *** *P* < 0.001. (**I**) Mug174 and Tgs1 are necessary for the TMG cap of U snRNAs. Purified small RNAs (input) and small RNAs precipitated utilizing an anti-TMG antibody were analyzed by northern blotting. (**J**) Mug174 associates with U2 and U5 snRNAs. Mug174-GFP, Tgs1-GFP and their parental untagged strains were subjected to RNA immunoprecipitation utilizing an anti-GFP antibody, and the precipitated fractions were assessed through RT-qPCR. Relative enrichment of U1, U2 and U5 snRNAs with *act1* RNA was computed and presented as mean ± S.D. (*n* = 3). * *P* < 0.05. (**K**) RNA-FISH using a Cy3-labeled U1, U2 or U5 snRNA specific probe in *S. pombe* cells expressing Mug174-GFP. The white dotted lines indicate the cell shapes. Scale bars, 10 μm.

We compared the primary sequence of Mug174 and Coilin proteins across other species. A multiple sequence alignment utilizing Clustal Omega suggested that the N- and C-terminal domains of Mug174 are weakly homologous to Coilin proteins (Supplementary Figure S4A and B), similar to the previous findings that Coilin proteins in higher eukaryotes show sequence similarities only in the N- and C-terminal domains throughout species (21,23). Intriguingly, a protein phylogenic tree suggested that the primary sequence is more extensively conserved between Mug174 and human Coilin than between fruit fly and human Coilins (Figure 4A). Notably, a recent structural prediction using AlphaFold claimed that Mug174 is a structural homolog of Coilin (Supplementary Figure S4C) (59). These findings are aligned with the idea that Mug174 is the fission yeast Coilin ortholog.

CBs facilitate RNA modifications (e.g. 5′ cap trimethylation and 2′-O-ribose methylation), and human Coilin (hCoilin) interacts with the trimethylguanosine synthase TGS1 and the box C/D snoRNP complex (20,60). To test our hypothesis that Mug174 should interact with these RNA-modifying enzymes, we performed a yeast two-hybrid (Y2H) assay and identified that Mug174 interacts with Tgs1/TGS1 under the most stringent (-Leu-His-Trp-Ade) conditions (Figure 4B). The relationship between endogenous Mug174 and Tgs1 was confirmed by co-immunoprecipitation followed by western blotting (Figure 4C). We attempted to identify the Mug174 domain required for the interaction with Tgs1 and identified that the N-terminal segment of Mug174 is necessary for the interaction (Supplementary Figure S4D). Given that this interaction was observed in a less stringent (-Leu-His-Trp) condition (Supplementary Figure S4D), the N-terminal domain is necessary but may not be sufficient for the strong Mug174 and Tgs1 interaction. Similar to these observations, fluorescence microscopic analyses demonstrated that Mug174 colocalized with Tgs1 in vegetative cells (Figure 4D). We also investigated the interaction between Mug174 and the box C/D snoRNP complex constituents (Fib1/FBL, Snu13/SNU13, Nop56/NOP56 and Nop58/NOP58), but we did not identify any direct association with the Y2H system (data not shown). Still, Mug174 partially colocalized with Fib1/FBL and Nop56/NOP56 (Supplementary Figure S4E), indicating a functional link between Mug174 and the box C/D snoRNP complex.

Cajal body integrity requires ongoing U snRNP biogenesis mediated by TGS1, as CBs are dispersed into multiple foci in the absence of TGS1 (61). Similarly, the absence of the *tgs1^+^* gene resulted in an increase in the number of Mug174 foci without affecting the steady-state level of Mug174 (Figure 4E and Supplementary Figure S4F and G). Furthermore, Tgs1 did not localize adequately in the absence of Mug174, while the steady-state level of the Tgs1 protein was not altered (Figure 4F and Supplementary Figure S4H and I). These findings suggest that the localization of Mug174 and Tgs1 is interdependent, demonstrating that Mug174 and Tgs1 are functionally and physically linked.

A primary function of CBs is to enable U snRNP maturation (20,25). As Mug174 is physically associated with Tgs1, we investigated whether Mug174 positively regulates the trimethylation of U snRNAs. We first conducted immunofluorescence using an anti-trimethylguanosine cap (TMG) antibody and observed a reduction in the TMG levels in *mug174*Δ and *tgs1*Δ (Figure 4G). TMG-immunoprecipitation followed by RT-qPCR demonstrated the loss of the TMG capping of U snRNAs, while the expression levels of U snRNAs were not altered in *mug174*Δ and *tgs1*Δ (Figure 4H and Supplementary Figure S4J). The absence of TMG capping of U snRNAs was also confirmed by RNA IP followed by northern blotting (Figure 4I). Moreover, RNA-immunoprecipitation experiments suggested that Mug174 was significantly bound to U2 and U5 snRNAs (Figure 4J), and RNA-FISH demonstrated that U2 snRNA signals coincided with Mug174 (Figure 4K). Additionally, RT-PCR demonstrated the ratio of unspliced/spliced mRNAs in *mug174*Δ*tgs1*Δ aligned with that in *mug174*Δ (Supplementary Figure S4K), and the growth of *mug174*Δ*tgs1*Δ was almost identical to *mug174*Δ (Supplementary Figure S4L and M). From these analyses, we found that Mug174 and Tgs1 operate in the same pathway, promoting U snRNA maturation through TMG capping.

We examined how hCoilin behaves in fission yeast. Interestingly, hCoilin-GFP formed nuclear foci when expressed in fission yeast and colocalized with Mug174-tdTomato (Supplementary Figure S4N), suggesting that both Mug174 and hCoilin produce nuclear condensates with similar physical properties. We also examined whether hCoilin expression could rescue the growth defect in *mug174*Δ, but it did not (Supplementary Figure S4O), suggesting that the primary sequence of hCoilin has diverged significantly from Mug174, and hCoilin cannot operate alongside *S. pombe* proteins.

According to these results, Mug174 is found to be the fission yeast ortholog of Coilin. Fission yeast also possesses Cajal body-like nuclear condensates, which had not been previously described.

### Chromosome segregation defects in *mug174*Δ

**Figure 5.**
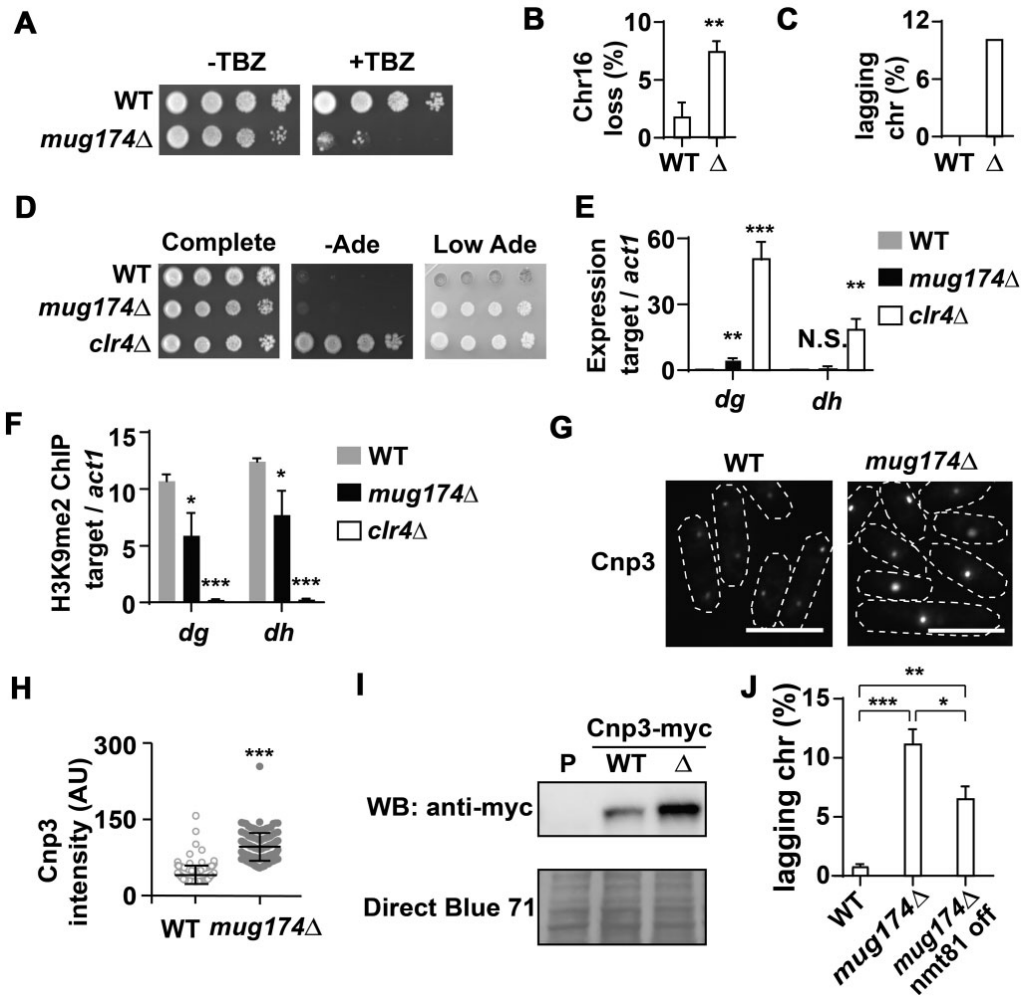
Mitotic chromosome segregation is adversely influenced in *mug174*Δ. (**A**) *mug174*Δ cells exhibit sensitivity to thiabendazole (TBZ). Ten-fold serial dilutions of wild-type (WT) and *mug174*Δ cultures were placed onto complete medium plates with or without TBZ (15 μg/ml) and incubated at 32°C for four days. (**B**) The loss of the minichromosome Ch16m23 (Ch16) in WT and *mug174*Δ. Over 1000 colonies were assessed, and the mean ± S.D. was presented (*n* = 3). ** *P* < 0.01. (**C**) Lagging chromosomes in the M phase of WT and *mug174*Δ. Over 100 M phase cells were examined across each strain. (**D**) Ten-fold serial dilutions of WT, *mug174*Δ and *clr4*Δ harboring the *ade6*^+^ marker gene inserted at centromere 1 (*otr1*R::*ade6*^+^) were placed onto complete, adenine-lacking (-Ade), and low adenine-containing (Low Ade) plates, and incubated at 32°C for four days. (**E**) RT-qPCR of *dg* and *dh* transcripts in WT, *mug174*Δ, and *clr4*Δ cells. The fold changes were normalized relative to the *act1* mRNA (mean ± S.D., *n* = 3). ** *P* < 0.01 and *** *P* < 0.001. N.S.: not significant. (**F**) Chromatin immunoprecipitation (ChIP) of H3K9me2 at *dg* and *dh* in WT, *mug174*Δ cells, and *clr4*Δ cells. Relative enrichment (mean ± S.D., *n* = 3) was determined from three-independent ChIP-qPCR experiments. The reference locus was *act1*^+^. * *P* < 0.05 and *** *P* < 0.001. (**G**) Cnp3-tdTomato localization in WT and *mug174*Δ cells. White dotted lines indicate the cell shapes. Scale bars, 10 μm. (**H**) A vertical scatter plot of Cnp3-tdTomato signal intensity in WT and *mug174*Δ cells. Over 100 cells were assessed. AU: arbitrary unit. (**I**) Western blotting of Cnp3-myc in WT and *mug174*Δ cells. The parental strain (P) was employed as a negative control. The protein loading was confirmed by Direct Blue 71 staining. (**J**) Lagging chromosomes in *mug174*Δ were partially limited by reducing the Cnp3 protein level. The *cnp3* mRNA expression was repressed using the thiamine-repressive promoter *nmt81*. * *P* < 0.05, ** *P* < 0.01 and *** *P* < 0.001.

Inactivation of Cajal body components is tied to various human diseases and impaired reproduction in mice (4,20,24), but it remains unclear how malfunctions of CBs produce such detrimental effects. To delve deeper into this question, we further characterized Mug174. A dilution analysis suggested that *mug174*Δ was more sensitive to thiabendazole (TBZ) than its parental WT strain (Figure 5A). TBZ is a microtubule-destabilizing agent, and chromosome segregation mutants (e.g. RNAi defective mutants) are sensitive to TBZ (62–64). We examined the stability of the minichromosome Ch16m23 (65) in mitotically dividing WT and *mug174*Δ cells. As expected, *mug174*Δ lost its minichromosome more frequently than WT (Figure 5B and Supplementary Figure S5A). Moreover, fluorescence microscopic analyses found lagging chromosomes in mitotic *mug174*Δ cells more frequently than in mitotic WT cells (Figure 5C and Supplementary Figure S5B). Overall, we conclude that Mug174 is required for proper mitotic chromosome segregation.

### Partial heterochromatin loss at centromeres in *mug174***Δ**

The loss of centromeric heterochromatin can trigger lagging chromosomes (62–64). As Tgs1 is involved in heterochromatin establishment (42), we anticipated that centromeric heterochromatin would be disrupted in *mug174*Δ. To assess the integrity of centromeric heterochromatin, we utilized *otr*1R::*ade6*^+^, an *ade6*^+^ maker gene in the pericentromeric heterochromatin of chromosome 1 (34). *ade6*^+^ de-repression can be assessed on plates containing low adenine according to the color (red: silenced; white: de-repressed) of colonies. We identified that *mug174*Δ cells formed whitish colonies on low-adenine plates (Figure 5D, Low Ade) and that not all *mug174*Δ colonies were white, whereas a fraction of them were pinkish in color (Supplementary Figure S5C). In the same assay, *clr4*Δ, lacking histone H3 Lys9 methylation, was grown on no adenine (–Ade) plates (Figure 5D), and the color of *clr4*Δ cells was completely white (Supplementary Figure S5C). These observations are aligned with our RNA-seq data that the centromeric transcript *SPNCRNA.234* was elevated most significantly, suggesting that centromeric silencing is partly compromised in *mug174*Δ. We conducted RT-qPCR and histone H3 Lys9 dimethylation (H3K9me2) chromatin immunoprecipitation (ChIP)-qPCR at two centromeric repetitive sequences, *dg* and *dh* (34). RT-qPCR demonstrated that *dg* but not *dh* transcripts were significantly increased in *mug174*Δ (Figure 5E), and ChIP-qPCR unveiled the levels of H3K9me2 at both *dg* and *dh* were significantly reduced in *mug174*Δ (Figure 5F). Collectively, these data indicate that Mug174 plays a minor role in the formation of centromeric heterochromatin, relative to the H3K9 methyltransferase Clr4/SUV39.

### Cnp3/CENP-C, a kinetochore protein, abnormally accumulates in *mug174*Δ

Unexpectedly, we identified that Cnp3/CENP-C foci are larger in *mug174*Δ than in WT (Figure 5G). We verified variability in Cnp3 signals between WT and *mug174*Δ by investigating a combination of the two strains under a fluorescent microscope (Supplementary Figure S5D). The quantification of Cnp3 signals demonstrated that the signal intensity of Cnp3 almost doubled with statistical significance in the absence of Mug174 (Figure 5H). Similar to this observation, we identified that the steady-state levels of the Cnp3 protein were elevated in *mug174*Δ relative to the WT (Figure 5I and Supplementary Figure S5E) and that Cnp3 accumulated at kinetochores in *mug174*Δ at higher levels than in WT (Supplementary Figure S5F). Conversely, *cnp3* mRNA levels in WT and *mug174*Δ were comparable (Supplementary Figure S5G). We also examined the localization of five kinetochore proteins (Cnp1/CENP-A, Cnp20/CENP-T, Mis12/MIS12, Ndc80/NDC80, and Spc7/KNL1). However, no alteration was observed in their signal intensity in *mug174*Δ (data not shown). These findings suggest that the steady-state level of the Cnp3 protein, but not other kinetochore proteins, is elevated, and more Cnp3 proteins are localized to kinetochores in the absence of Mug174. As Cnp3 overexpression causes lagging chromosomes (66), we speculated that lagging chromosomes in *mug174*Δ are partially due to increased Cnp3 protein. To examine this, we replaced the endogenous *cnp3*^+^ promoter with the thiamine-repressive *nmt81* promoter (67). In the presence of thiamine, Cnp3 expression and Cnp3 intensity were significantly reduced (Supplementary Figure S5H-K). As depicted in Figure 5J, repressed Cnp3 expression suppressed lagging chromosomes in *mug174*Δ. While it remains unclear how Mug174 modulates the steady-state level of Cnp3, we conclude that Mug174 is necessary for proper kinetochore assembly.

### Mug174/Coilin is necessary for cellular quiescence

**Figure 6.**
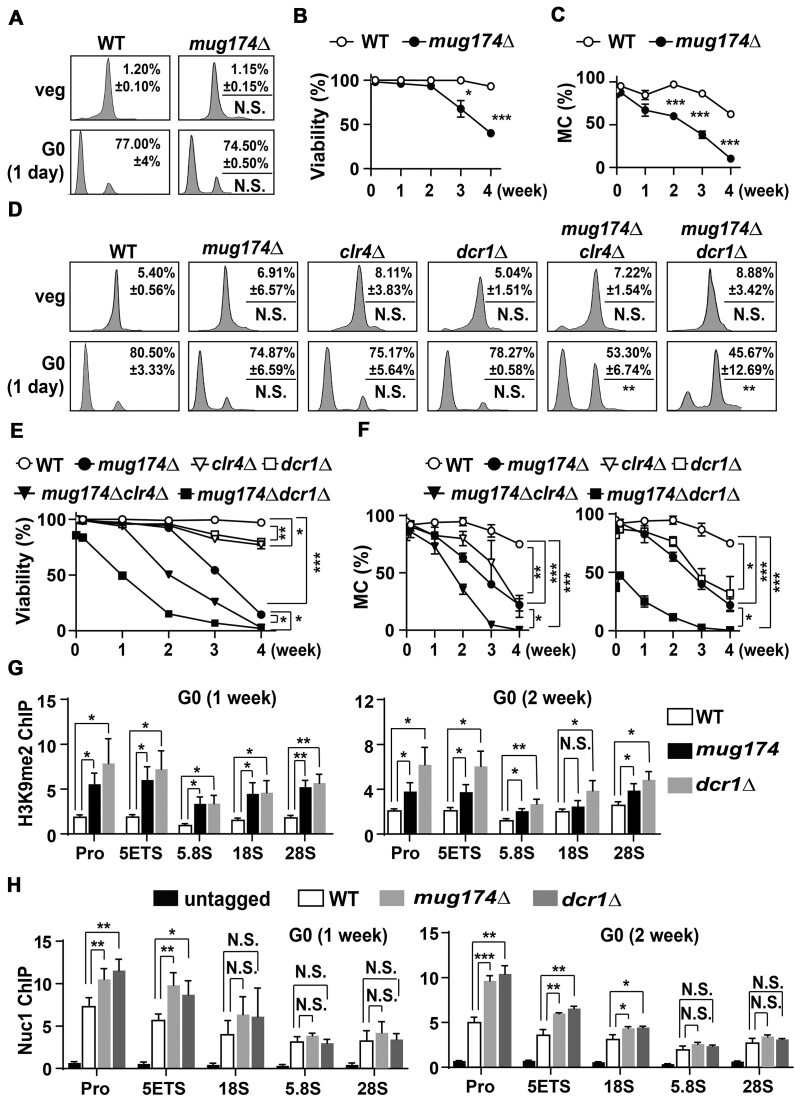
Mug174/Coilin is required for cellular quiescence. (**A**) G0 entry in wild-type (WT) and *mug174*Δ upon nitrogen starvation was assessed by flow cytometry during vegetative growth (veg) and 1 day following nitrogen depletion (G0). N.S.: not significant. (**B**) Cell viability in WT and *mug17*4Δ cells following nitrogen starvation. Trypan blue exclusion analysis was conducted to test cell viability, and over 200 cells were examined. The mean ± S.D. was presented from three independent experiments. * *P* < 0.05 and *** *P* < 0.001. (**C**) Mitotic competence (MC), the ability to revert to mitotic division from G0, of WT and *mug174*Δ cells over various time points following nitrogen deprivation. Three independent experiments were performed to characterize the mean ± S.D., and 90 cells were examined each time. *** *P* < 0.001. (**D**) G0 entry in WT, *mug174*Δ, *clr4*Δ, *dcr1*Δ, *mug174*Δ*clr4*Δ and *mug174*Δ*dcr1*Δ upon nitrogen starvation was examined via flow cytometry during vegetative growth (veg) and 1 day following nitrogen depletion (G0). ** *P* < 0.01. N.S.: not significant. (E and F) G0 cell viability (**E**) and the mitotic competence (**F**) of the indicated strains after G0 induction. Three independent experiments were performed to determine the mean ± S.D., and over 120 cells were examined each time. * *P* < 0.05, ** *P* < 0.01 and *** *P* < 0.001. (**G**) The H3K9me2 levels at the rDNA locus in WT, *mug174*Δ and *dcr1*Δ G0 cells. Relative enrichment (mean ± S.D.) was characterized by five independent ChIP-qPCR experiments using G0 cells following 1 week and 2 weeks of nitrogen starvation. The reference locus was *act1*^+^. * *P* < 0.05 and ** *P* < 0.01. N.S.: not significant. (**H**) The Nuc1 levels identified at the rDNA locus in WT, *mug174*Δ and *dcr1*Δ following 1 week and 2 weeks of nitrogen starvation. Relative enrichment (mean ± S.D.) was identified using four-independent ChIP-qPCR experiments. The reference locus was *act1*^+^. * *P* < 0.05 and ** *P* < 0.01. N.S.: not significant.

Our characterization indicated that *mug174*Δ is sensitive to cold and defective in meiosis, suggesting that CBs (or Mug174) facilitate adaptations to environmental changes. Nitrogen deprivation induces G0/cellular quiescence in prototrophic, heterothallic *S. pombe* strains (68). We hypothesized that G0 is disturbed without functional CBs (or Mug174). To investigate this, we measured the entry into the G0 phase in WT and *mug174*Δ cells. Fluorescence-activated cell sorting (FACS) analyses of nitrogen-starved prototrophic WT and *mug174*Δ cells indicated that both WT and *mug174*Δ cells entered the G0 phase appropriately (Figure 6A), demonstrating that Mug174 is dispensable for G0 entry. We then investigated cell viability during the G0 phase through trypan blue staining. WT cells maintained high cell viability for four weeks following nitrogen depletion (Figure 6B), as documented previously (69). However, *mug174*Δ began to lose cell viability three weeks following nitrogen starvation, and >50% of *mug174*Δ cells were inviable four weeks following G0 induction (Figure 6B). These observations suggest that Mug174/Coilin is required to maintain cell viability during cellular quiescence. We assessed the mitotic competence (the ability to revert to the mitotic cycle from G0) of *mug174*Δ, identifying that WT cells maintained high mitotic competence three weeks after nitrogen deprivation, and over 50% of WT cells still reverted to the mitotic cycle four weeks after G0 entry (Figure 6C). Conversely, the mitotic competence of *mug174*Δ G0 cells began to decrease significantly after two weeks, with most *mug174*Δ G0 cells losing mitotic competence after 4 weeks (Figure 6C). According to these findings, we conclude that Mug174/Coilin is required for maintaining cell viability during cellular quiescence and reverting to vegetative growth.

### Mug174/Coilin and heterochromatin assembly factors contribute differently to cellular quiescence

Prior studies have demonstrated that RNA interference (RNAi) and Clr4/SUV39, both critical for heterochromatin assembly in *S. pombe*, have essential roles in G0 (70,71). As *mug174*Δ cells are partially defective in heterochromatin formation, Mug174 may operate in the same pathway as Clr4 and RNAi (e.g. Dcr1/DICER). To investigate this possibility, we combined *mug174*Δ with either *clr4*Δ or *dcr1*Δ and examined the G0 entry and cell viability of the double deletion strain. When combining *mug174Δ* with *dcr1*Δ via genetic crossing, we determined that *mug174*Δ*dcr1*Δ cells grew slower than WT, *mug174*Δ, or *dcr1*Δ during vegetative growth (Supplementary Figure S6A), indicating that Mug174 and Dcr1 work in distinct or overlapping pathways in mitotically dividing cells. When G0 was induced, *mug174*Δ*clr4*Δ and *mug174*Δ*dcr1*Δ did not efficiently enter G0 phase, while WT, *mug174*Δ, *clr4*Δ, and *dcr1*Δ strains did (Figure 6D). In addition, the loss of both G0 cell viability and mitotic competence was more substantial in the double deletion strains compared to WT, *mug174*Δ, *clr4*Δ or *dcr1*Δ (Figure 6E and F). Consistent with the G0 defects, we found enlarged cells and those lacking DAPI staining, particularly in nitrogen-starved *mug174*Δ*clr4*Δ and *mug174*Δ*dcr1*Δ (Supplementary Figure S6B). This finding suggests that these abnormally shaped cells are dead or extremely ill. From these data, we determine that Mug174 and Clr4/Dcr1 operate in distinct or overlapping pathways in cellular quiescence.

### Mug174/Coilin prevents aberrant histone H3 Lys9 methylation and RNA pol I occupancy at rDNA repeats

We investigated how Mug174 maintains cell viability and mitotic competence in quiescent cells. Aberrant H3K9me2 accumulation at rDNA repeats during G0 caused cell death in *dcr1*Δ (70). This report prompted us to investigate H3K9me2 at rDNA in quiescent *mug174*Δ cells. We performed H3K9me2 ChIP-qPCR on rDNA (Supplementary Figure S6C) in WT, *mug174*Δ, and *dcr1*Δ cells during vegetative growth and in the G0 phase. A significant elevation in H3K9me2 on rDNA repeats in *mug174*Δ as well as *dcr1*Δ was observed 1 and 2 weeks after G0 induction (Figure 6G). Conversely, we did not observe an increase during vegetative growth or 1 day after G0 induction (Supplementary Figure S6D). This suggests that abnormal H3K9me2 accumulation occurs in *mug174*Δ, although it was not as clear as in *dcr1*Δ. A previous study demonstrated that G0 defects in *dcr1*Δ are suppressed by *clr4*Δ (70). As illustrated in Figure 6E and F, *clr4*Δ did not limit the loss of cell viability and mitotic competence in *mug174*Δ. These data confirm the idea that Mug174 and Dcr1 have specific roles in G0 cells and indicate that the aberrant H3K9me2 increase at rDNA repeats does not produce G0 defects in *mug174*Δ.

Another potential explanation is that Mug174 limits Nuc1/POLR1A (RNA polymerase I subunit A) occupancy at rDNA as Dcr1 evicts Pol I from rDNA to maintain cell viability in G0 (70). We examined RNA Pol I occupancy at rDNA using ChIP-qPCR. No significant variations in Pol I occupancy at rDNA were observed in WT, *mug174*Δ, and *dcr1*Δ cells during vegetative growth and 1 day following G0 induction (Supplementary Figure S6E). In contrast, the occupancy of Nuc1 at the promoter (Pro), 5′ external transcribed spacer sequences (5′ETS), and 18S rDNA was increased in *mug174*Δ during prolonged (1 and 2 weeks) G0 phases, and the influence of *mug174*Δ on Pol I occupancy was similar to *dcr1*Δ (Figure 6H). This finding suggested that the increased Pol I occupancy may cause heightened transcript numbers from rDNA repeats. We assessed 28S and 18S rRNA levels, which were significantly higher in *mug174*Δ following 1 and 2 weeks of nitrogen starvation (Supplementary Figure S6F). Additionally, GO analyses of whole-cell proteome analyses indicated that the proteins linked to ribosome biogenesis were upregulated in *mug174*Δ 1 and 2 weeks following G0 induction (Supplementary Tables S11–S13). Since rRNA levels rapidly decrease upon G0 induction (43), the increased levels of rRNA transcripts and ribosome-related proteins found in *mug174*Δ may adversely impact quiescent cells.

### mRNA splicing defects negatively influence cellular quiescence

**Figure 7.**
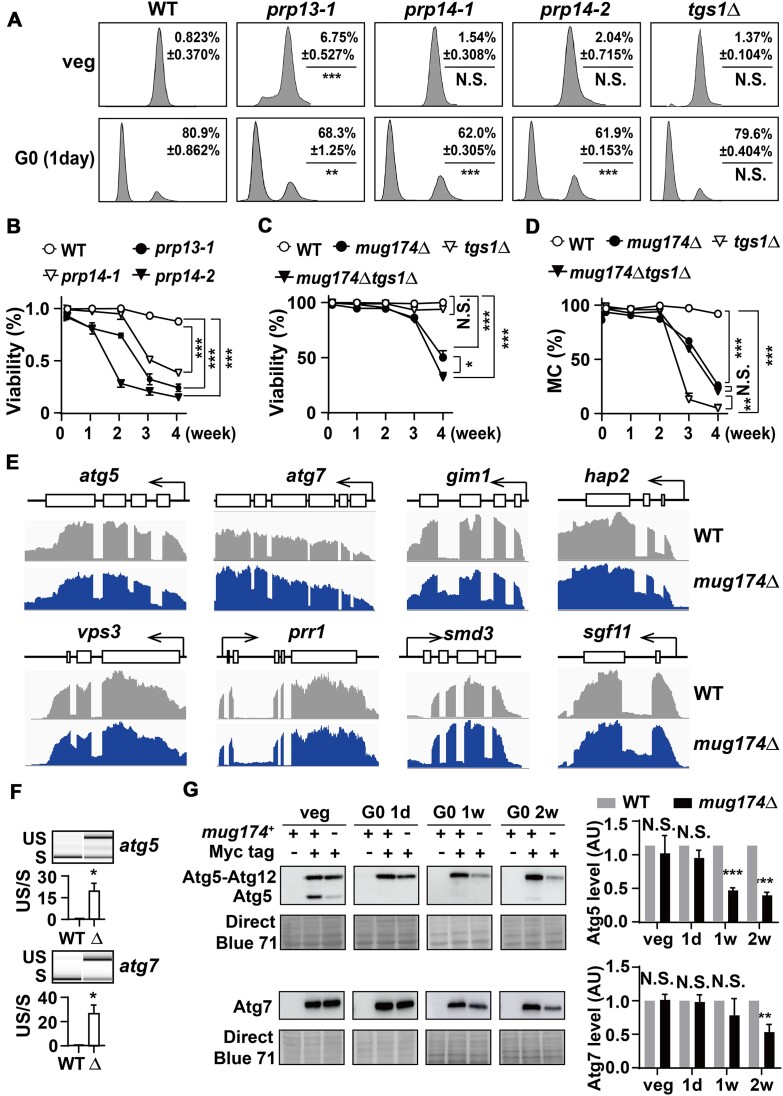
The necessity of mRNA splicing factors in cellular quiescence. (**A**) G0 entry in wild-type (WT), *prp13-1*, *prp14-1*, *prp14-2* and *tgs1*Δ upon nitrogen starvation was examined by flow cytometry during vegetative growth (veg) and 1 day following nitrogen depletion. (**B**) G0 cell viability in WT, *prp13-1*, *prp14-1* and *prp14-2* cells following nitrogen starvation. Trypan blue exclusion analysis was conducted to assess cell viability, and over 200 cells were examined. The mean ± S.D. was determined from three independent experiments. *** *P* < 0.001. (**C**) G0 cell viability in WT, *mug174*Δ, *tgs1*Δ and *mug174*Δ*tgs1*Δ was examined using trypan blue staining at the indicated time points. *** *P* < 0.05 and *** *P* < 0.001. N.S.: not significant. (**D**) The mitotic competence (MC) of WT, *mug174*Δ, *tgs1*Δ and *mug174*Δ*tgs1*Δ cells at various time points following nitrogen deprivation. Three independent experiments were conducted to calculate the mean ± S.D., and 182 cells were examined. ** *P* < 0.01 and *** *P* < 0.001. N.S.: not significant. (**E**) Strand-specific RNA-seq read coverage of the G0 essential (GZE) genes in WT (gray) and *mug174*Δ (blue) cells. Exons and the direction of transcription are depicted as open boxes and arrows, respectively. (**F**) RT-PCR results of the exon-exon junctions of the two representative genes in WT and *mug174*Δ strains. The lower bands indicate the spliced (S) transcripts, while the upper bands represent the unspliced (US) transcripts of the indicated genes. The ratio of spliced (S) versus unspliced (US) mRNAs of *atg5*^+^ (top) and *atg7*^+^ (bottom) was quantified (mean ± S.D., *n* = 3). * *P* < 0.05. (**G**) (left) Western blotting of Atg5-myc and Atg7-myc in both vegetative (veg) and G0 (1 day, 1 week, and 2 weeks following nitrogen starvation) WT and *mug174*Δ cells. Their parental strain was employed as a negative control for western blotting. The protein loading was assessed by Direct Blue 71 staining. (right) The Atg5 or Atg7 band intensity in WT and *mug174*Δ was characterized via three independent western blotting results, and the mean ± S.D. was presented. AU: arbitrary unit. ** *P* < 0.01 and *** *P* < 0.001. N.S.: not significant.

Given that Mug174 is necessary for appropriate pre-mRNA splicing, the G0 defects identified in *mug174*Δ could be due to mRNA splicing defects. To investigate this idea, we examined whether Prp13/U4 snRNA (72) or Prp14/DHX38, which catalyzes the first transesterification reaction of spliceosomal mRNA splicing (73), is necessary for cell viability during cellular quiescence. Both Prp13 and Prp14 are required for cell viability, and we used the mutant strains, *prp13-1*, *prp14-1* and *prp14-2*, which show splicing defects at permissive temperatures (72,74). We identified that G0 entry via FACS analysis was significantly reduced in *prp13-1*, *prp14-1* and *prp14-2* cells (Figure 7A). Moreover, the G0 cell viability of the three *prp* mutants declined relative to the WT (Figure 7B). These findings suggest that proper mRNA splicing is crucial for G0 entry and G0 cell viability, but the *prp* mutants and *mug174*Δ exhibit different phenotypes, as G0 entry was unaltered by *mug174*Δ (Figure 6A).

Recent studies have determined that Tgs1/TGS1 is required for mRNA splicing in both *S. pombe* and fruit flies (42,75). Given that Tgs1 interacts and colocalizes with Mug174, it may be involved in cellular quiescence. FACS analyses indicated that Tgs1 is dispensable for typical G0 entry (Figure 7A). Unexpectedly, the G0 cell viability of *tgs1*Δ was indistinguishable from WT (Figure 7C). In addition, mitotic competence was more severely impacted in *tgs1*Δ than in *mug174*Δ or *mug174*Δ*tgs1*Δ (Figure 7D), suggesting that Tgs1 is vital for mitotic competence maintenance during cellular quiescence but not for G0 viability. The variability between *mug174*Δ and *tgs1*Δ implies that Mug174 has an additional function outside cap trimethylation.

Overall, we conclude that mRNA splicing is necessary for cellular quiescence, as well as the mitotic cell cycle, but each splicing factor contributes differently to G0 entry and maintaining G0 cell viability and mitotic competence.

### Mug174/Coilin supports the expression of G0 essential (GZE) genes

The mRNA splicing machinery could be necessary for the expression of genes associated with the maintenance of G0 viability and mitotic competence, including G0 essential (GZE) genes (76). RNA-seq findings suggested that intronic signals of eight GZE genes were increased in *mug174*Δ (Figure 7E). The RNA-seq data of *atg5* and *atg7* mRNAs were confirmed via RT-PCR (Figure 7F), and the expression levels of the two GZE proteins, Atg5 and Atg7, essential for autophagy (77), were reduced in *mug174*Δ (Figure 7G). The reduction in protein expression was negatively correlated with the increase in intron accumulation (Figure 7E). These data support the necessity of pre-mRNA splicing for cellular quiescence in fission yeast.

## Discussion

Our localization-based approach has found various proteins forming discrete nuclear condensates in *S. pombe*. In this study, we characterized Mug174 via biochemical, cell biological, and genetic approaches and demonstrated its requirement for cell growth, chromosome segregation, mRNA splicing, snRNA maturation, cellular quiescence, and meiotic processes. According to our findings, Mug174 is the fission yeast ortholog of Coilin, a necessary component of the Cajal body, and that *S. pombe* has a Cajal body-like nuclear structure, which has not been described previously in fission yeast. Therefore, we anticipate that our study using a unicellular eukaryote offers additional insights into the functions of Coilin, an enigmatic protein, in higher eukaryotes (21).

### Coilin proteins and Cajal bodies are conserved throughout lower and higher eukaryotes

Prior studies have reported that Coilin proteins exist in multicellular organisms like fruit flies, zebrafish, mice, and humans (21,23). However, Coilin has not previously been described in either *S. cerevisiae*, *S. pombe* or *C. elegans* prior to this study. Our characterization of Mug174 suggested that it is functionally equivalent to Coilin, and supported by a recent protein structural prediction by AlphaFold (59). Therefore, we expect *S. cerevisiae* and *C. elegans* to also have Coilin orthologs. *S. cerevisiae* has no Mug174/Coilin homolog based on our BLAST search (unpublished observation); however, it also has a nuclear compartment called the nucleolar body, where U3 snoRNP matures (78). Intriguingly, a standard BLAST search indicated that Mug174 homologs are present across several *Caenorhabditis* species, including *C. japonica*, *C. nigoni* and *C. remenai*, but not in *C. elegans* (unpublished observation). From these findings, we believe that CBs are evolutionarily conserved from yeast to humans, and investigating Mug174 homologs in worms may yield additional information to better comprehend CBs in multicellular organisms.

### A functional link between the Cajal body and the cleavage body

Our protein localization analyses uncovered that CBs are often associated with cleavage bodies (CLBs), where mRNA polyadenylation factors are enriched (79). This linkage between two nuclear condensates is supported by physical interactions between Mug174 and the cleavage body component Red1, essential for RNA surveillance in the nuclear exosome (37,80). Moreover, the relationship between CBs and CLBs in human cells has also been reported (81,82). Based on these results, we speculate that CBs are functionally linked to CLBs, for instance, through the regulation of snRNA or snoRNA transcription. Previous integrative analyses demonstrated that CBs physically bind to sn/snoRNA genes, arranging those loci in transcriptionally active intra/inter-chromosomal regions in human cells (83). In *S. pombe*, Red1 binds to most snoRNA gene loci (58). Therefore, both CBs (or Coilin) and CLBs (or Red1) may enable the transcription of specific genes (e.g. snoRNAs) by modulating genome conformation. Alternatively, the interaction of CBs and CLBs may enable the coordination of snoRNA transcription by Coilin and snoRNA transcription termination by Red1 (58).

### Coilin: a conserved player for centromeres?

We demonstrated that Mug174 is associated with assembling pericentromeric heterochromatin. Because the intimate linkages between RNA splicing and heterochromatin assembly have been outlined in *S. pombe* (72,74,81,84–87), Mug174 is likely to operate as an RNA splicing factor. However, we cannot exclude the possibility that Mug174 possesses an additional function for heterochromatin assembly. In *Arabidopsis thaliana*, the AGO4-Pol IV-siRNA complex, essential for RNA-directed heterochromatin formation and DNA methylation, is assembled in CBs (19,88,89). It would be interesting to assess whether Coilin has a specific function in heterochromatin formation and whether hCoilin is a prerequisite for pericentromeric heterochromatin.

We revealed the abnormal accumulation of Cnp3/CENP-C at kinetochores in *mug174*Δ (Figure 5). The elevated Cnp3 intensity reminded us of the nucleoporin Alm1, necessary for promoting the degradation of Cnp3 via the proteasome (66). Our preliminary data strongly suggested that Mug174 allows Cnp3 ubiquitination, but Mug174 and Alm1 do not operate in the same pathway (unpublished observations). It is conceivable that Mug174 is necessary for the splicing of pre-mRNAs encoding Cnp3 degradation factors (the *alm1*^+^ gene contains no intron). However, based on previous studies, Mug174 likely acts directly on kinetochores (or Cnp3 proteins). For instance, overexpression of Annexin A2, a calcium-dependent phospholipid-binding protein, results in the degradation of CENP-A/C in a Coilin-dependent manner (90). Additionally, Coilin localization to kinetochores has been documented in humans and fruit flies (23,91). Additional experiments are necessary to confirm the evolutionary conservation of the role of Coilin on kinetochore proteins.

### *S. pombe* Coilin is a potential regulator that handles environmental stresses

Previous studies reported that *mug174*^+^ is a gene upregulated in meiosis, and Mug174 is essential for proper sporulation (46,48). Consistent with these findings, our study demonstrated that Mug174 is necessary for efficient mating, sporulation, and spore viability. Similarly, lower fertility was observed in mice without Coilin (24), implying the importance of CBs for gametogenesis. In addition to meiosis, Mug174 enhances cell viability during quiescence and assists quiescent cells in re-entering the mitotic cell cycle. We suspect that defects in cellular quiescence caused by a malfunction of CBs cause human diseases (4). Both meiosis and cellular quiescence are induced by nitrogen deprivation in fission yeast (92,93). Additionally, we identified that *mug174*Δ is sensitive to cold. These findings indicate that CBs assist in adaptations to environmental alterations directly and indirectly. Consistent with this hypothesis, multiple stresses, encompassing heat/cold shock, serum starvation, and osmotic stress, influence Cajal body organization in higher eukaryotes (94), suggesting that CBs respond to differences in environmental conditions. Additionally, plant Coilin is likely a sensor of certain viral proteins and promotes salicylic acid-mediated anti-viral protection by trapping poly(ADP-ribose) polymerase in the nucleolus (16). Therefore, examining the potential role(s) of CBs in sensing or adapting to environmental changes is meaningful.

However, the mechanism(s) by which CBs (or Coilin) operate upon environmental changes remains unclear. One possible mechanism for normal G0 is that CBs promote appropriate mRNA splicing, as splicing mutants were defective in G0 entry and maintaining G0 viability. Another possible mechanism is the suppression of RNA polymerase I (Pol I) transcription by Coilin. In fission yeast, Pol I is evicted from rDNA regions in response to G0 induction as Pol I transcription in G0 is deleterious (70). Notably, Coilin suppresses Pol I transcription by removing Pol I from rDNA during DNA damage in human cells (21). Similar to this report, we observed increased Pol I occupancy following G0 induction in *mug174*Δ. We speculate that Coilin promotes G0 viability through Pol I suppression and mRNA splicing.

### Perspective

*S. pombe* cells lacking Mug174 exhibited multiple phenotypes, including G0 and chromosome segregation defects. Given that *mug174*Δ adversely affected pre-mRNA splicing, we acknowledge the potential that the various phenotypes we identified in *mug174*Δ are simply due to incomplete splicing of mRNAs encoding essential regulators for distinct biological pathways, as outlined above. Still, these phenotypes identified in *mug174*Δ are informative in speculating how Cajal body malfunctions result in various symptoms, including bone marrow failure, neurodegeneration, and immunodeficiency (4,95). One possibility is that cellular quiescence, essential for the viability of long-lived cells (96), is disrupted without functional CBs, affecting long-lived cells like stem, neuronal, and immune cells. We hope that further examinations of CBs will provide potential clues for developing therapeutic approaches for diseases resulting from the malfunctioning of CBs.

## Supplementary Material

gkae463_Supplemental_Files

## Data Availability

The data supporting this study are available from the corresponding authors upon reasonable request. The accession numbers of the RNA-seq datasets described in this paper is GEO: GSE246256. The mass spectrometry datasets have been deposited to the ProteomeXchange Consortium via PRIDE with identifier PXD052066.
