# Supplementary material for: The fission yeast ortholog of Coilin, Mug174, forms Cajal body-like nuclear condensates and is essential for cellular quiescence
Source: Nucleic Acids Res. 2024 Jun 3;52(15):9174–92. doi: 10.1093/nar/gkae463 (PMC11347179; doi:10.1093/nar/gkae463)
Supplement: gkae463_Supplemental_Files [file gkae463_supplemental_files.zip › 240503_Supplementary Table Legends.docx]

**Supplementary table legends**

**Table S1.** Strains used in this study, related to Experimental Procedures

**Table S2.** Primers used in this study

**Table S3.** Plamids used in this study

**Table S4.** Genes showing increased expression (*mug174*Δ/WT_logFC > 0.6), related to figure 4

**Table S5.** Genes showing decreased expression (*mug174*Δ/WT_logFC <-0.6), related to figure 4

**Table S6.** GO analysis of genes differentially expressed in vegetative cells

**Table S7.** Mass spectrometry results of upregulated (*mug174*Δ/WT_logFC > 0.6) proteins in *mug174*Δ vegetative cells, related to figure 4

**Table S8.** Mass spectrometry results of downregulated (*mug174*Δ/WT_logFC <-0.6) proteins in *mug174*Δ vegetative cells, related to figure 4

**Table S9.** GO analysis of proteins differentially expressed vegetative cells

**Table S10.** Overlap of differentially expressed genes and proteins

**Table S11.** Mass spectrometry results of differentially expressed proteins in *mug174*Δ G0 1 week cells

**Table S12.** Mass spectrometry results of differentially expressed proteins in *mug174*Δ G0 2 week cells

**Table S13.** GO analysis of differentially expressed proteins in *mug174*Δ G0 1 week or 2 week cells

**Supplementary movie legend**

**Movie S1.** Spherical droplets formed by SNAP-Mug174
