# Supplementary material for: The fission yeast ortholog of Coilin, Mug174, forms Cajal body-like nuclear condensates and is essential for cellular quiescence
Source: Nucleic Acids Res. 2024 Jun 3;52(15):9174–92. doi: 10.1093/nar/gkae463 (PMC11347179; doi:10.1093/nar/gkae463)
Supplement: gkae463_Supplemental_Files [file gkae463_supplemental_files.zip › 240523_corrected Supplementary Figures.pdf]

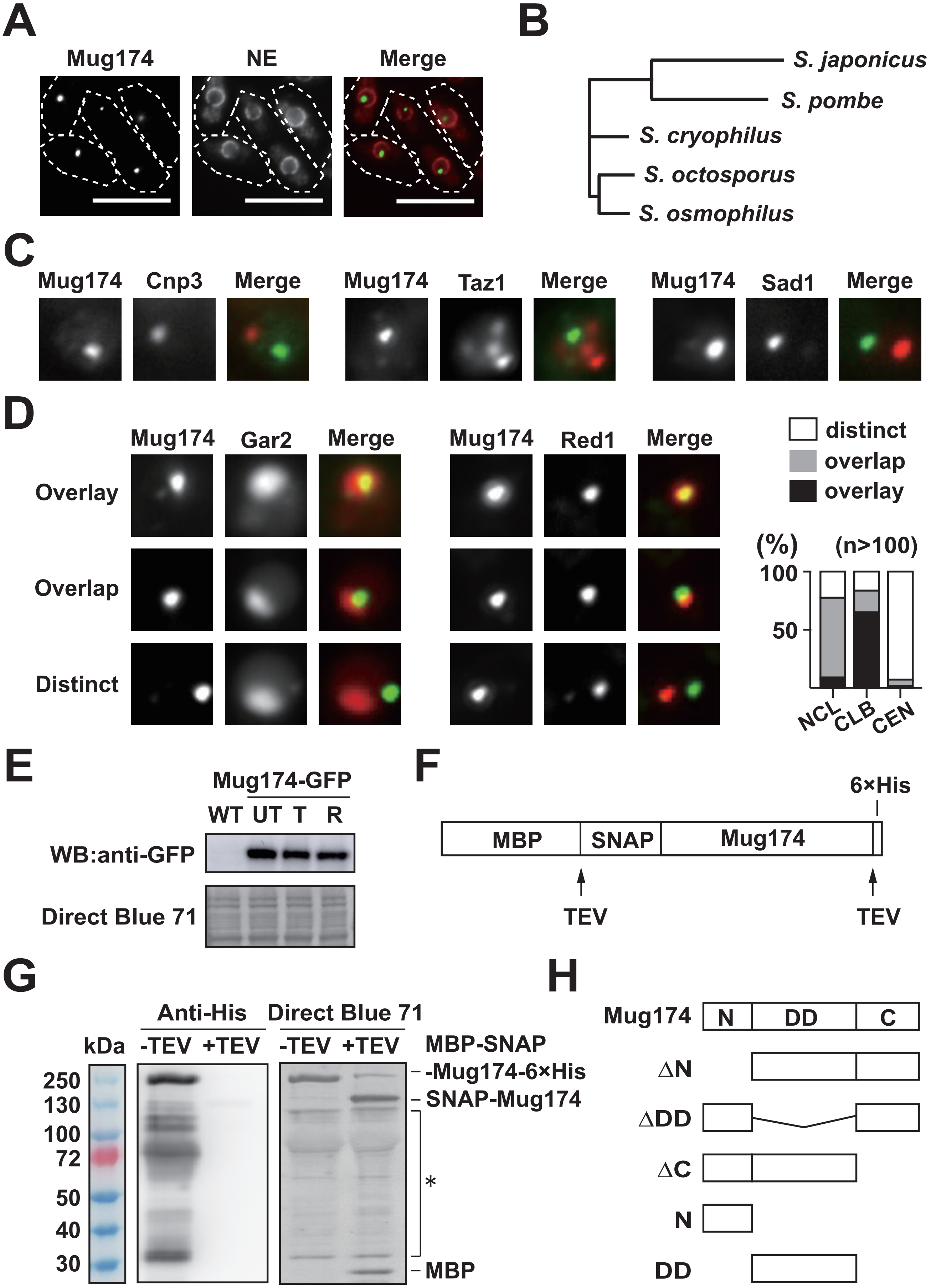
A
Mug174
NE
Merge
B
S. japonicus
S. pombe
S. cryophilus
S. octosporus
S. osmophilus
C
Mug174
Cnp3
Merge
Mug174
Taz1
Merge
Mug174
Sad1
Merge
D
Mug174
Gar2
Merge
Mug174
Red1
Merge
Overlay
Overlap
Distinct
distinct
overlap
overlay
(%)
(n>100)
100
50
NCL
CLB
CEN
E
Mug174-GFP
WT
UT
T
R
WB:anti-GFP
Direct Blue 71
F
6×His
MBP
SNAP
Mug174
TEV
TEV
G
Anti-His
Direct Blue 71
kDa
-TEV
+TEV
-TEV
+TEV
MBP-SNAP
-Mug174-6×His
SNAP-Mug174
250
130
100
72
50
40
30
*
MBP
H
Mug174
N
DD
C
ΔN
ΔDD
ΔC
N
DD

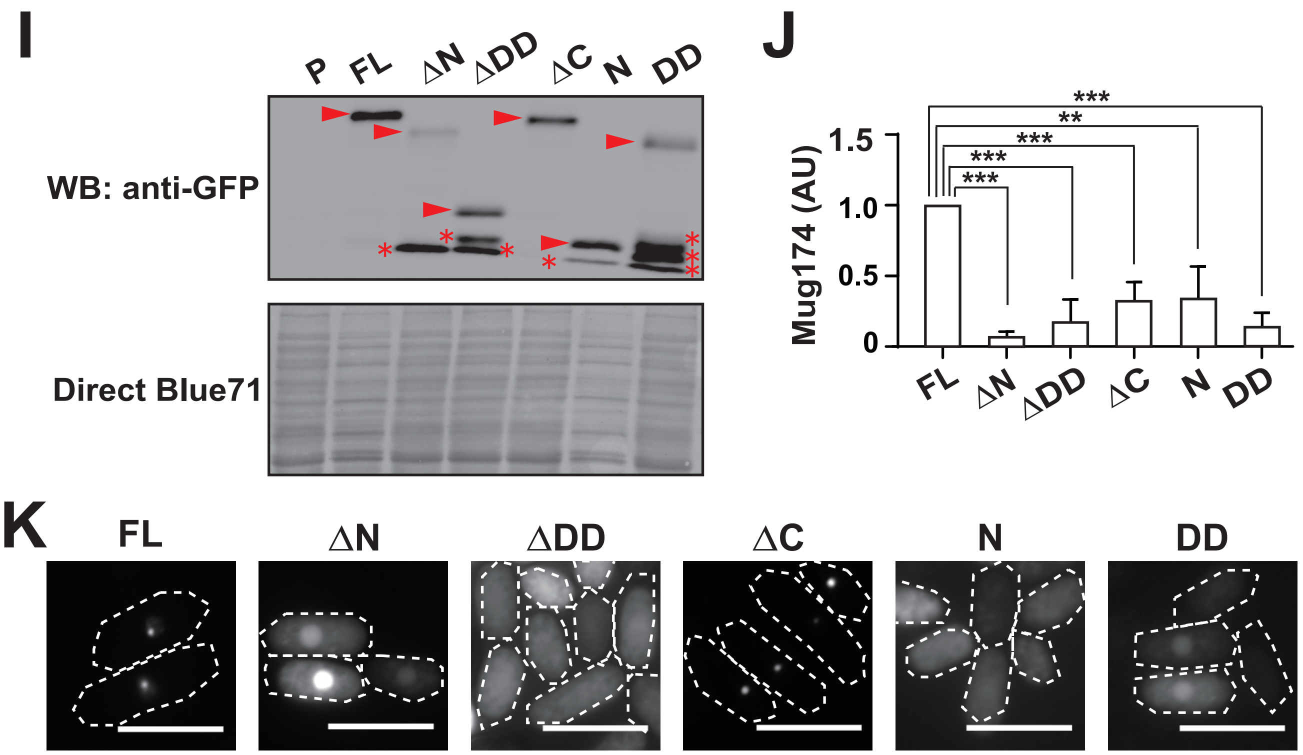

Supplementary Figure S1. Mug174 amino acid sequences and their expression.

(A) Localization of Mug174 and the nuclear membrane protein Cut11/NDC1. A strain expressing Mug174-GFP and Cut11-3×RFP was examined by fluorescence microscopy. The white dotted lines denote the cell shapes. Scale bars, 10 µm. NE: nuclear envelope. (B) A phylogenic tree of the Mug174 proteins found in the five Schizosaccharomyces species (*S. pombe*, *S. cryophilus*, *S. japonicus*, *S. octosporus*, and *S. osmophilus*) using Clustal Omega. (C) Magnified images of the nuclear region of cells expressing Mug174-GFP/Cnp3-tdTomato, Mug174-GFP/Taz1-tdTomato, or Mug174-tdTomato/Sad1-GFP. (D) Magnified images of the nuclear area of cells expressing Mug174-GFP/Gar2-mCherry or Mug174-tdTomato/Red1-GFP. The frequency of overlap or overlay between Mug174 and the nucleolus (NCL), cleavage body (CLB), or centromeres (CEN). (Right) The bar graph indicates the percentage of complete (overlay, black), partial (overlap, gray), or no colocalization (distinct, white) between Mug174 and either Gar2 (NCL), Red1 (CLB), or Cnp3 (CEN). More than 100 cells were examined. (E) Steady-state levels of Mug174-GFP before (UT), during (T), and after (R) 1,6-hexanediol treatment were assessed via western blotting, and protein loading was assessed by Direct Blue 71 staining of the blotted membrane. WT: a protein sample from an untagged strain, serving as a negative control. (F) A schematic representation of MBP-SNAP-Mug174-6×His. Two TEV protease cleavage sites are also depicted. (G) Purified MBP-SNAP-Mug174-6×His expressed in *E. coli* (-TEV) was subjected to TEV cleavage (+TEV) to eliminate both MBP and His tags. Purified proteins were examined on SDS-PAGE gels, and subjected to western blotting via probing with an anti-His tag antibody or stained with Direct Blue 71. As purified Mug174 protein is unstable in vitro, we observed degradation products. *: degraded fragments of MBP-SNAP-Mug174-6×His. (H) A schematic representation of Mug174 constructs: Mug174 full length, ΔN, ΔDD, ΔC, N, and DD. (I) Western blotting of GFP-fused Mug174 proteins. Red arrowheads and asterisks denote the target bands and the bands corresponding to degraded proteins, respectively. The protein loading was assessed using Direct Blue 71. (J) The quantification of various Mug174 proteins according to western blotting results. $**p < 0.01$ and $***p < 0.001$. (K) Localization of various Mug174-GFP fusion proteins [Full length (FL), ΔN, ΔDD, ΔC, N, and DD] expressed exogenously in vegetative cells. White dotted lines indicate the cell shapes. Scale bars, 10 µm.

A

Chromosome	Localization in genome	Gene	extent of rescue
2	4377265 - 4386682	*trp1*, *nop16*, *chr4*, *uap2*	strong
3	110632 - 118881	*mug135*, *ura4*, *new25*	strong
3	1773546 - 1777778	*PCC1442.04c*, *mic26*	weak
3	2001928 - 2007238	*ptc1*	weak
3	1997589 - 2006026	*ams2*, *ptc1*	weak
3	974200 - 983797	*sum3*, *svp26*	weak
1	1519823 - 1530296	*sec21*, *SPAC57A7.09*, *pzh1*	weak
1	1525318 - 1536038	*pzh1*, *SPAC57A7.07c*, *utp14*	weak
1	5237661 - 5247743	*nab2*, *SPAC14c4.07*, *mug5*, *agn1*, *SPAC14c4.10c*	weak
2	622000 - 630023	*rpc31*, *rpl1601*, *efm4* , *tef103*	weak
1	3303343 - 3311393	*glt1*	weak
2	1100203 - 1109409	*SPBC1709.03*, *cyp3*, *sks2*	weak

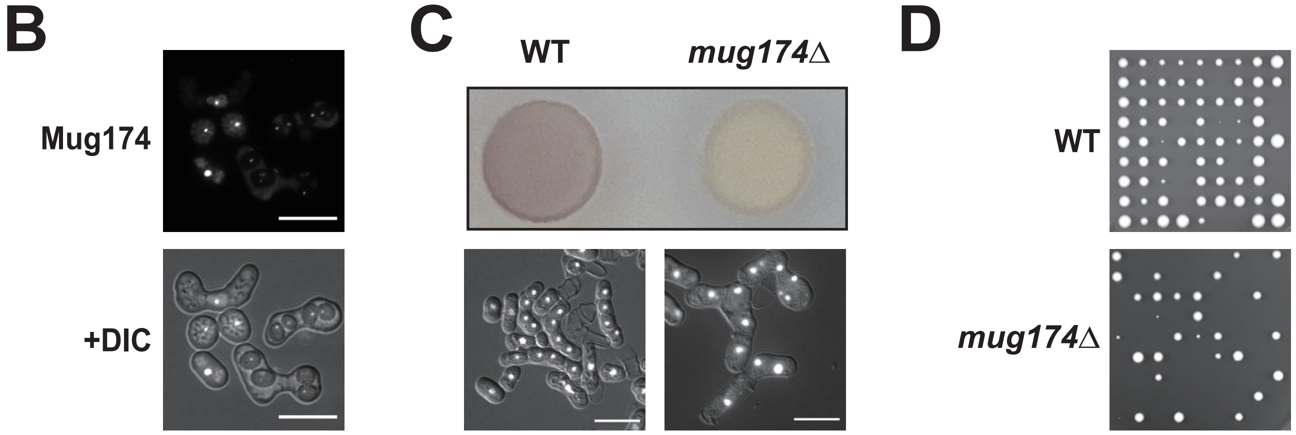

Supplementary Figure S2. Characterization of Mug174 roles in mitotic and meiotic phases.
(A) A list of the multi-copy suppressors of *mug174*Δ. The origin of each genomic DNA fragment (chromosome and nucleotides), genes in each genomic DNA fragment, and the extent of rescue are illustrated. (B) Localization of Mug174 throughout meiosis. Meiosis was induced in homothallic Mug174-GFP cells via nitrogen starvation, and cells at different meiotic stages are depicted. Scale bars, 10 µm. (C) Sporulation of WT and *mug174*Δ cells. (Top) Iodine staining of homothallic WT and *mug174*Δ cells on a sporulation plate. Homothallic WT and *mug174*Δ cells were cultivated on a sporulation plate and exposed to iodine vapor, staining enriched starch in spore walls. (Bottom) The DIC images of WT and *mug174*Δ asci. DNA was counterstained using Hoechst 33342. Scale bars, 10 µm. (D) Spores from homothallic WT and *mug174*Δ strains were plated on complete medium plates using a dissection microscope and incubated at 32°C.

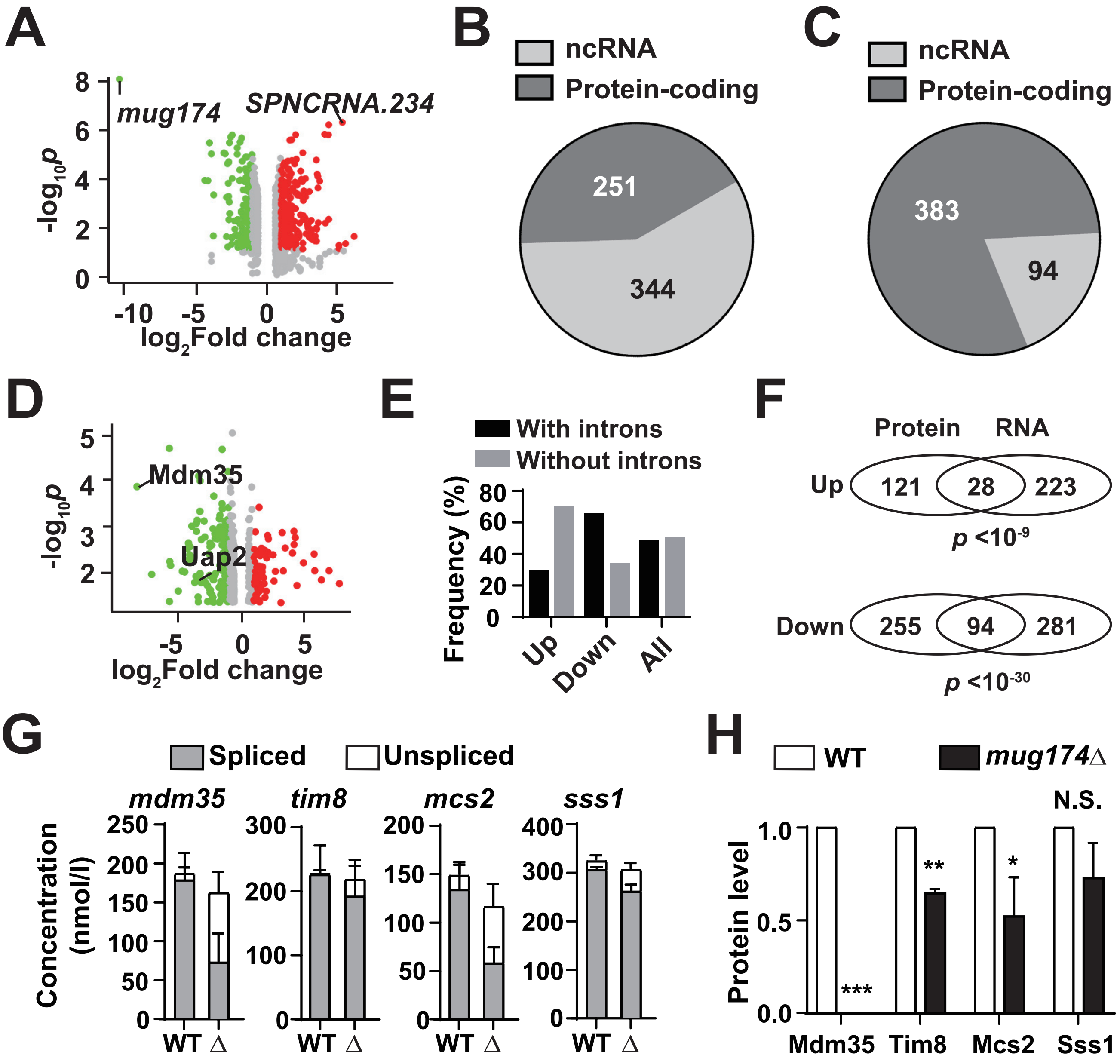

Supplementary Figure S3. Transcriptome alterations in *mug174*Δ.
(A) RNA-seq data indicating *mug174*Δ vs. wild-type (WT) are presented as a volcano plot depicting the statistical significance (Y-axis) versus fold changes (X-axis). Both significantly upregulated ($p < 0.05$, fold change $> 2^{0.6}$) and downregulated ($p < 0.05$, fold change $< 2^{-0.6}$) genes are identified by red and green dots, respectively. The remaining genes are depicted as gray dots. (B) A pie chart of the upregulated genes identified in *mug174*Δ. The elevated transcripts encompass 251 protein-coding genes and 344 ncRNA genes. (C) A pie chart of the downregulated genes identified in *mug174*Δ. The reduced transcripts are separated into 383 protein-coding genes and 94 ncRNA genes. (D) Mass spectrometry data of *mug174*Δ vs. WT are presented in a volcano plot depicting the statistical significance (y-axis) versus fold changes (x-axis). Both significantly upregulated ($p < 0.05$, fold change $> 2^{0.6}$) and downregulated ($p < 0.05$, fold change $< 2^{-0.6}$) proteins are identified by red and green dots, respectively. The remaining genes are depicted as gray dots. (E) Bar graph illustrating gene features (genes with introns and genes lacking introns) of differentially expressed proteins in whole cell proteome analyses. Up: genes encoding upregulated proteins; Down: genes encoding downregulated proteins; and All: all protein-coding genes. (F) The overlap of differentially expressed genes and proteins. Up-regulated group: representation factor: 3.8, $p < \sim 10^{-9}$. Down-regulated group: representation factor: 3.6, $p < \sim 10^{-30}$. (G) The spliced (gray) and unspliced (white) mRNA levels of the four (continued)

intron-containing genes in vegetative WT and *mug174*Δ (Δ) were depicted as a bar graph (mean ± S.D., n = 3). (H) The expression levels of four proteins encoded by intron-containing genes in vegetative WT and *mug174*Δ strains. The expression level of each protein in WT was characterized as 1 and used to determine that of each protein in *mug174*Δ. * p <0.05, ** p < 0.01, and *** p < 0.001. N.S.: not significant.

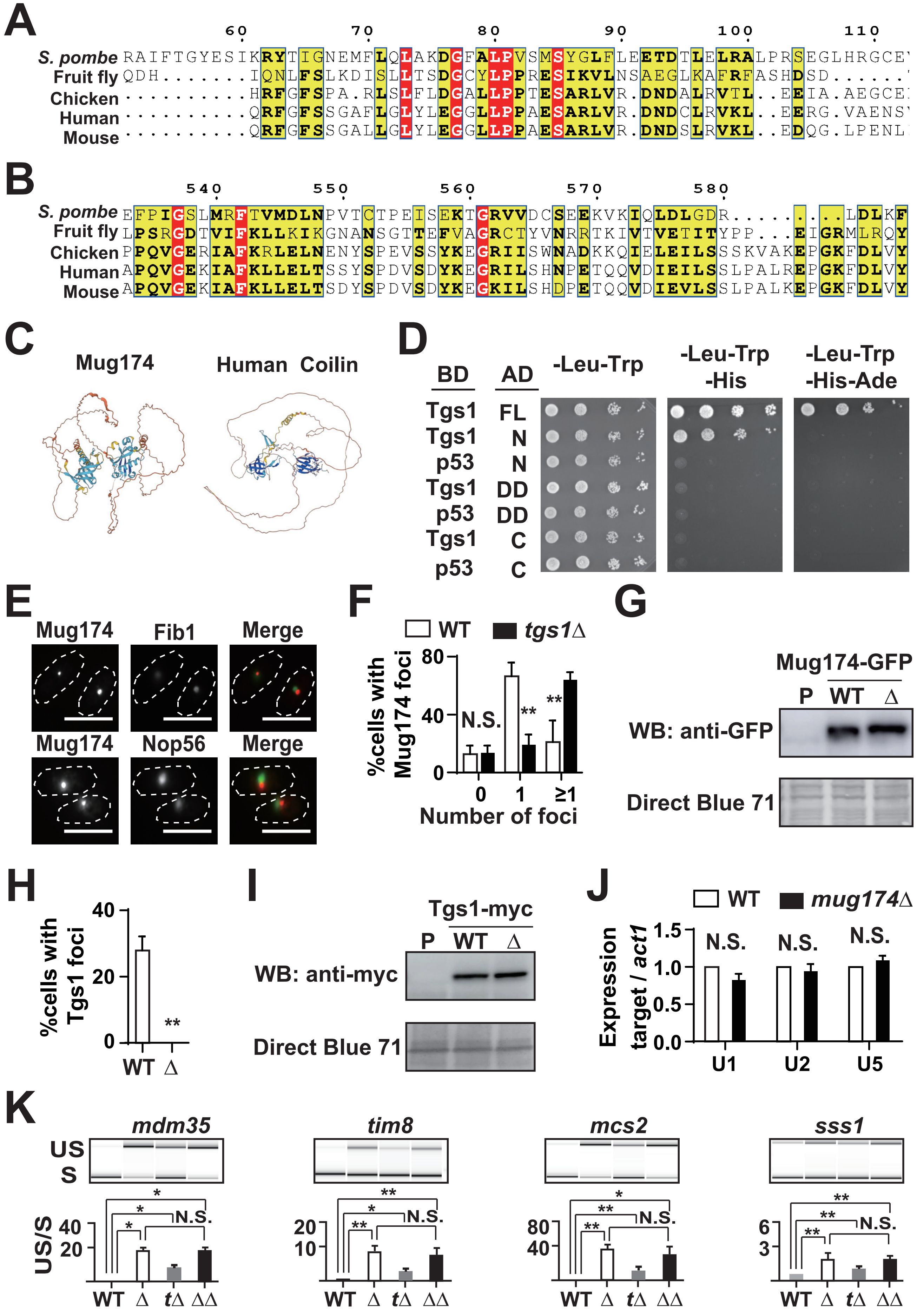
A
60
70
80
90
100
110
S. pombe RAIFTGYESIKRYTIGNEMFLQLAKDGFALPVSMSYGLFLEETDTLELRALPRSEGLHRGCE
Fruit fly QDH.......IQNLFSLKDISLLTSDGCYLPPRESIKVLNSAEGLKAFRFASHDSD......
Chicken ..........HRFGFSPA.RLSLFLDGALLPPTESARLVR.DNDALRVTL..EEIA.AEGCE
Human ..........QRFGFSSGAFLGLYLEGGLLPPAESARLVR.DNDCLRVKL..EERG.VAENS
Mouse ..........QRFGFSSGALLGLYLEGGLLPPAESARLVR.DNDSLRVKL..EDQG.LPENL
B
540
550
560
570
580
S. pombe EFPIGSLMRFTVMDLNPVTCTPEISEKTGRVVDCSEEKVKIQLDLGDR..........LDLKF
Fruit fly LPSRGDTVIFKLLKIKGNANSGTTEFVAGRCTYVNRRTKIVTVETITYPP...EIGRMLRQY
Chicken PPQVGERIAFKRLELNENYSPEVSSYKEGRIISWNADKKQIELEILSSSKVAKEPGKFDLVY
Human APQVGEKIAFKLLELTSSYSPDVSDYKEGRILSHNPETQQVDIEILSSLPALREPGKFDLVY
Mouse APQVGEKIAFKLLELTSDYSPDVSDYKEGKILSHDPETQQVDIEVLSSLPALKEPGKFDLVY
C
Mug174
Human Coilin
D
BD
AD
-Leu-Trp
-Leu-Trp -His
-Leu-Trp -His-Ade
Tgs1 FL
Tgs1 N
p53 N
Tgs1 DD
p53 DD
Tgs1 C
p53 C
E
Mug174
Fib1
Merge
Mug174
Nop56
Merge
F
WT
tgs1Δ
%cells with Mug174 foci
80
40
0
N.S.
**
**
0
1
≥1
Number of foci
G
Mug174-GFP
P
WT
Δ
WB: anti-GFP
Direct Blue 71
H
%cells with Tgs1 foci
40
20
0
**
WT
Δ
I
Tgs1-myc
P
WT
Δ
WB: anti-myc
Direct Blue 71
J
WT
mug174Δ
Expression target / act1
1.5
1.0
0.5
0.0
N.S.
N.S.
N.S.
U1
U2
U5
K
mdm35
tim8
mcs2
sss1
US
S
US/S
40
20
10
80
6
3
*
**
N.S.
WT
Δ
tΔ
ΔΔ

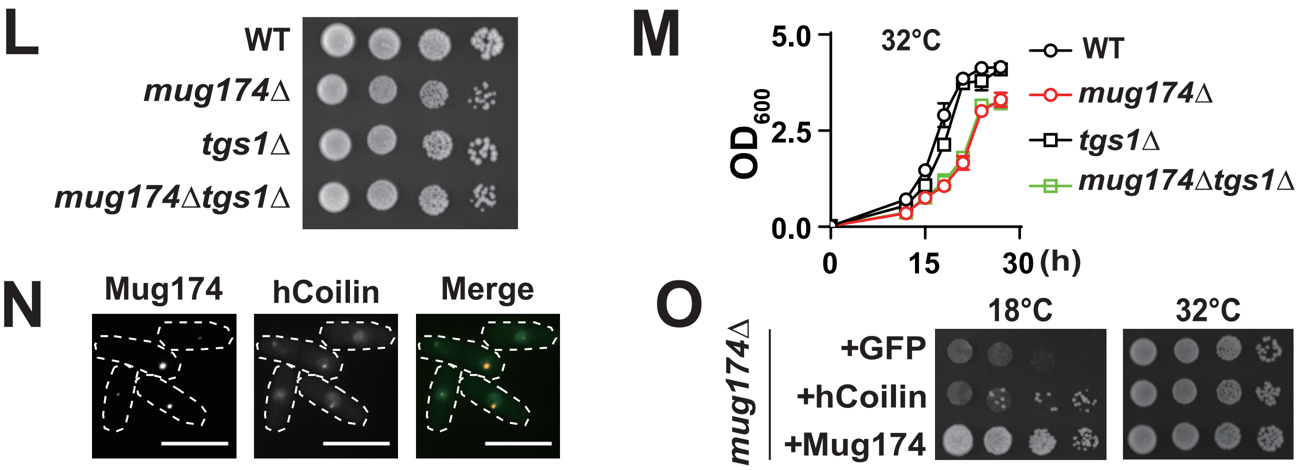

Supplementary Figure S4. Verification of Mug174 as the fission yeast ortholog of Coilin. (A and B) A multiple sequence alignment of Mug174 and Coilin proteins from indicated multicellular organisms. The N-terminal (A) and the C-terminal (B) domains, but not the disordered domain, exhibit sequence similarity. Red: identical amino acid residues; yellow: similar or well-conserved amino acid residues. (C) Predicted protein structures from Mug174 and human Coilin. The images are acquired from the AlphaFold Protein Structure Database (Supplementary references 1, 2). (D) The interactions between domains of Mug174 and Tgs1 were assessed using a standard yeast two-hybrid system. The full length of Mug174 (FL), the N-terminal domain (N), the intrinsically disordered domain (DD), and the C-terminal domain (C) were fused to the GAL4 activation domain (AD), while Tgs1 was fused to the GAL4 binding domain (BD). The combination of p53 and N, p53 and DD, and p53 and C served as negative controls. (E) Mug174 partially colocalizes with Fib1 (top panels) and Nop56 (bottom panels). Strains expressing Mug174-tdTomato and Fib1-GFP or Mug174-tdTomato and Nop56-GFP were assessed by fluorescence microscopy. The white dotted lines denote cell shapes. Scale bar, 10 μm. (F) The number of Mug174 foci in wild-type (WT) or *tgs1*Δ was counted. Over 100 cells were assessed three times and classified into three groups: none, one, and more than one dot. The mean ± S.D. (n = 3) was presented. $^{**}p < 0.01$. N.S.: not significant. (G) Western blotting of Mug174-GFP in WT and *tgs1*Δ (Δ) cells. Their parental untagged strain (P) served as a negative control. The protein loading was investigated by Direct Blue 71 staining of the blotted membrane. (H) The percentage of cells exhibiting the Tgs1 nuclear dot(s) in WT or *mug174*Δ. Over 100 cells were assessed three times, and the mean ± S.D. (n = 3) for each sample was depicted. $^{**}p < 0.01$. (I) Western blotting of Tgs1-myc in WT and *mug174*Δ (Δ) cells. Their parental untagged strain (P) operated as a negative control. The protein loading was identified via Direct Blue 71 staining of the blotted membrane. (J) U1, U2, and U5 snRNA expression in WT and *mug174*Δ were examined by RT-qPCR. Fold changes of U1, U2, and U5 snRNA expression in *mug174*Δ compared to WT cells were characterized and presented as mean ± S.D. (n = 3). N.S.: not significant. (K) RT-PCR findings of the exon-exon junctions in WT, *mug174*Δ (Δ), *tgs1*Δ (*t*Δ), and *mug174*Δ*tgs1*Δ (ΔΔ) cells. The ratios (mean ± S.D., n = 3) of unspliced (US) and spliced (S) fragments of genes were characterized and normalized relative to WT. $^{*}p < 0.05$ and $^{**}p < 0.01$. N.S.: not significant. (L) Dilution analysis of cell growth. Ten-fold dilutions of WT, *mug174*Δ, *tgs1*Δ, and *mug174*Δ*tgs1*Δ cells were plated onto a complete medium plate and grown at 32°C. (M) The growth curves of WT (open circle), *mug174*Δ (red circle), *tgs1*Δ (open box), and *mug174*Δ*tgs1*Δ (green box). All strains were cultured in complete liquid media at 32°C, and OD600 was measured at the indicated time points. (N) Colocalization of human Coilin-GFP and Mug174-tdTomato in *S. pombe*. Fission yeast cells expressing Mug174-tdTomato and human Coilin-GFP (hCoilin) were assessed by fluorescence microscopy. The white dotted lines indicate the cell shapes. Scale bar, 10 μm. (O) Human Coilin did not rescue the growth defect in *mug174*Δ. Ten-fold serial dilutions of *mug174*Δ integrated with human Coilin-GFP (+hCoilin), Mug174-GFP (+Mug174), or empty plasmid (+GFP) were plated onto complete medium plates and incubated at the indicated temperatures.

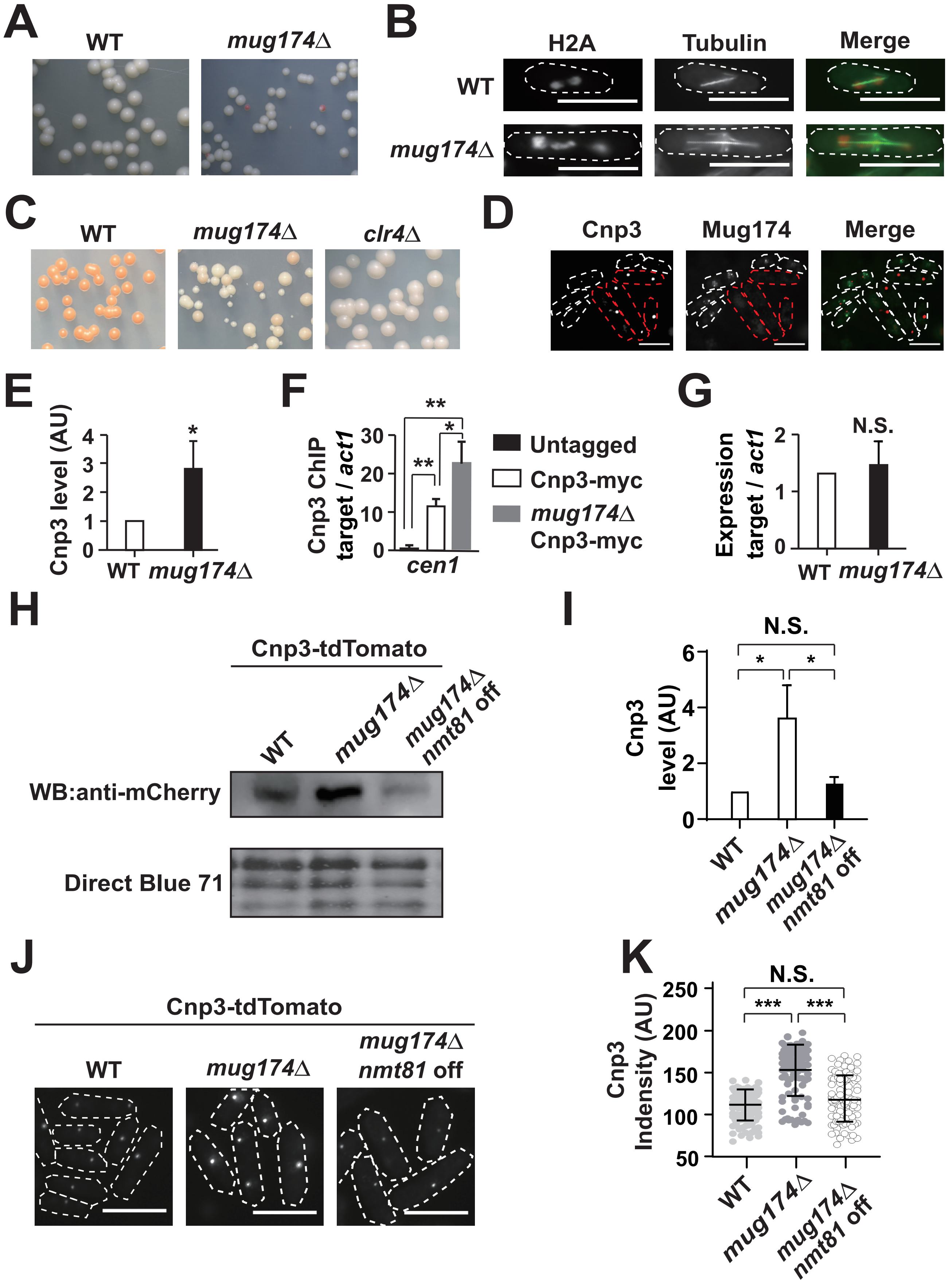

Supplementary Figure S5. Mug174 is required for proper chromosome segregation.
(A) Wild-type (WT) and *mug174*Δ cells carrying the minichromosome Ch16m23 were plated onto low adenine medium plates, and the plates were incubated at 32°C for 5 days. (continued)

(B) An example of lagging chromosomes in *mug174Δ*. Tubulin and chromosomes were identified using GFP-Atb2 (tubulin alpha 2) and Hta1 (histone H2A)-mCherry, respectively. The white dotted lines indicate cell shapes. Scale bars, 10 µm. (C) WT, *mug174Δ*, and *clr4Δ* carrying the *ade6*$^+$ marker gene at centromere 1 (*otr1R*::*ade6*$^+$) were plated on low adenine-containing plates, and incubated at 32°C for 4 days. (D) Cnp3-tdTomato localization in WT and *mug174Δ* cells. The two strains were combined prior to fluorescence microscopy. The white and red dotted lines depict WT and *mug174Δ*, respectively. To distinguish the two strains, Mug174-GFP expressed only in WT was used. Scale bars, 10 µm. (E) The quantification of Cnp3-myc intensity in *mug174Δ* compared to WT strains (mean ± S.D., n = 3) was presented as a bar graph. AU: arbitrary unit. *$p < 0.05$. (F) Cnp3-myc enrichment at the kinetochore region of chromosome I in WT, *mug174Δ*, and their parental untagged strain was investigated via ChIP-qPCR. The mean ± S.D. are presented (n = 3). *$p < 0.05$ and **$p < 0.01$. (G) RT-qPCR of *cnp3* mRNA in WT and *mug174Δ* strains. The *cnp3* mRNA expression relative to *act1* mRNA in WT was established as 1 and employed to determine the fold change of *cnp3* mRNA in *mug174Δ*. The mean ± S.D. are presented (n = 3). N.S.: not significant. (H) Western blotting of Cnp3-tdTomato in WT, *mug174Δ*, and *mug174Δ* with reduced Cnp3 expression (*mug174Δ nmt81* off) cells. The protein loading was examined by Direct Blue 71 staining of the blotted membrane. (I) The quantification of Cnp3-tdTomato intensity in *mug174Δ* or *mug174Δ* with reduced Cnp3 expression (*mug174Δ nmt81* off) relative to WT strains (mean ± S.D., n = 3) was presented as a bar graph. AU: arbitrary unit. *$p < 0.05$. N.S.: not significant. (J) Cnp3-tdTomato localization in WT, *mug174Δ*, and *mug174Δ* with reduced Cnp3 expression (*mug174Δ nmt81* off) cells. White dotted lines indicate cell shapes. Scale bars, 10 µm. (K) A vertical scatter plot of Cnp3-tdTomato signal intensity in WT, *mug174Δ*, and *mug174Δ* with reduced Cnp3 expression (*mug174Δ nmt81* off) cells. Over 100 cells were assessed. AU: arbitrary unit.

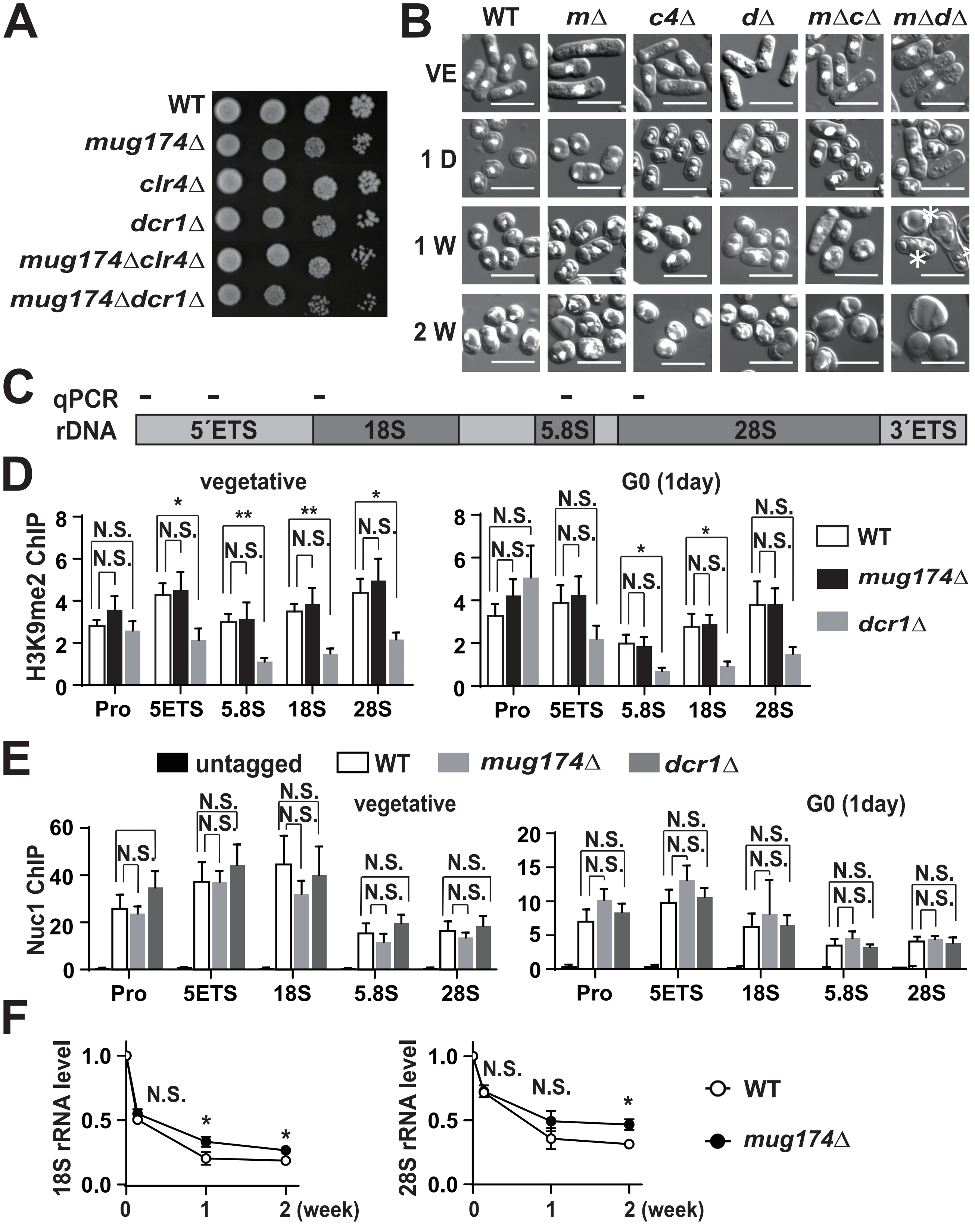

Supplementary Figure S6. Characterization of Mug174 in cellular quiescence.
(A) Dilution analysis of wild-type (WT), *mug174*Δ, *clr4*Δ, *dcr1*Δ, *mug174*Δ*clr4*Δ, and *mug174*Δ*dcr1* Δ. Ten-fold serial dilutions of the indicated cells were plated onto a complete medium plate and incubated at 30°C. (B) The DIC images of WT, *mug174*Δ, *clr4*Δ, *dcr1*Δ, *mug174*Δ*clr4*Δ, and *mug174*Δ *dcr1*Δ at the indicated time points. *Cells lacking DAPI signal. (C) A schematic representation of the rDNA structure. The small bars denote the target DNA fragments assessed by qPCR. (continued)

(D) The H3K9me2 levels at the rDNA locus in vegetative WT, *mug174*Δ, and *dcr1*Δ cells and G0 cells 1 day after nitrogen-starvation. Relative enrichment (mean ± S.D.) was assessed from five independent ChIP-qPCR experiments. The reference locus was $act1^+$. *p < 0.05 and **p < 0.01. N.S.: not significant. (E) The Nuc1 levels at the rDNA locus in WT, *mug174*Δ, and *dcr1*Δ during vegetative growth (veg cells) and G0 cells following 1 day of nitrogen-starvation. Relative enrichment (mean ± S.D.) was determined according to four independent ChIP-qPCR experiments. The reference locus was $act1^+$. N.S.: not significant. (F) 28S and 18S rRNA levels in each cell were measured. Total RNAs isolated from WT and *mug174*Δ after 0, 1, 7, and 14 days of G0 induction were examined using an Agilent Genetic Analyzer 2100. *p < 0.05. N.S.: not significant.

Supplementary references

1. Jumper, J., Evans, R., Pritzel, A., Green, T., Figurnov, M., Ronneberger, O., Tunyasuvunakool, K., Bates, R., Žídek, A., Potapenko, A. *et al.* (2021) Highly accurate protein structure prediction with AlphaFold. *Nature*, **596**, 583-589.
2. Varadi, M., Bertoni, D., Magana, P., Paramval, U., Pidruchna, I., Radhakrishnan, M., Tsenkov, M., Nair, S., Mirdita, M., Yeo, J. *et al.* (2024) AlphaFold Protein Structure Database in 2024: providing structure coverage for over 214 million protein sequences. *Nucleic Acids Res*, **52**, D368-D375.
